# Versatile functional roles of horizontal cells in the retinal circuit

**DOI:** 10.1038/s41598-017-05543-2

**Published:** 2017-07-17

**Authors:** Taro Chaya, Akihiro Matsumoto, Yuko Sugita, Satoshi Watanabe, Ryusuke Kuwahara, Masao Tachibana, Takahisa Furukawa

**Affiliations:** 1 Laboratory for Molecular and Developmental Biology, Institute for Protein Research, Osaka University, JST, CREST, 3-2 Yamadaoka, Suita, Osaka 565-0871 Japan; 2 Department of Psychology, Graduate School of Humanities and Sociology, The University of Tokyo, JST, CREST, 7-3-1 Hongo, Bunkyo-ku, Tokyo 113-0033 Japan; 3 Research Center for Ultrahigh Voltage Electron Microscopy, Osaka University, 7-1 Mihogaoka, Ibaraki, Osaka 567-0047 Japan

## Abstract

In the retinal circuit, environmental light signals are converted into electrical signals that can be decoded properly by the brain. At the first synapse of the visual system, information flow from photoreceptors to bipolar cells is modulated by horizontal cells (HCs), however, their functional contribution to retinal output and individual visual function is not fully understood. In the current study, we investigated functional roles for HCs in retinal ganglion cell (RGC) response properties and optokinetic responses by establishing a HC-depleted mouse line. We observed that HC depletion impairs the antagonistic center-surround receptive field formation of RGCs, supporting a previously reported HC function revealed by pharmacological approaches. In addition, we found that HC loss reduces both the ON and OFF response diversities of RGCs, impairs adjustment of the sensitivity to ambient light at the retinal output level, and alters spatial frequency tuning at an individual level. Taken together, our current study suggests multiple functional aspects of HCs crucial for visual processing.

## Introduction

The vertebrate retina consists of five major classes of neurons that form highly organized neural circuits. Horizontal cells (HCs) and amacrine cells (ACs), two types of retinal interneurons, modulate the information flow from photoreceptors (PRs) to bipolar cells (BCs) in the outer plexiform layer (OPL) and from BCs to retinal ganglion cells (RGCs) in the inner plexiform layer (IPL), respectively. Although critical roles for ACs in visual information processing have been well characterized, our knowledge about the functional contribution of HCs is still limited^1, 2^. HCs, electrically coupled to each other by gap junctions, receive glutamatergic input from PRs and provide feedback and feedforward signals to PRs and BCs, respectively^3, 4^. Three different inhibitory feedback mechanisms have been proposed: GABA-mediated feedback^5–7^, hemichannel-mediated ephaptic feedback^8–11^, and pH-mediated feedback^12–16^. By combining two-photon microscopy and pharmacological treatment, Kemmler *et al*. indicated that cone output may be modulated directly by hemichannel-mediated ephaptic and pH-mediated feedback, and indirectly by GABA^17^. Although the role of HCs in antagonistic center-surround RF organization has been characterized by pharmacological approaches, how extensively HCs contribute to visual information processing and perception *in vivo* is not yet fully understood.

Animal models depleted in a certain neuronal cell type have been used to analyze the *in vivo* function of the targeted cell type in the retina^18–20^ as well as in the brain^21, 22^. Several mouse lines lacking retinal HCs have been generated previously. Loss of HCs in the developing stage causes defects in the retinal layer structure as well as in PR ribbon synapse formation^23–25^. To eliminate the consequences of the absence of HCs in developing stages, a mouse line in which HCs were depleted after the completed formation of the retinal neural circuit was reported^26^. Depletion of HCs at the adult stage in this mouse line leads to rod PR cell death and ectopic synapse formation in the outer nuclear layer (ONL) probably due to long-term removal of HCs in the retina. These histrogical changes were observed at the stage when electrophysiological and behavioral analyses were performed^26^.

In the current study, we established and analyzed HC-depleted (dHC) mice to investigate HC functions. Although we observed that HCs are totally fragmented in the dHC retina, there were no significant differnces in the ONL and total layer thickness between control and dHC retinas. We did not observe ectopic synapse formation in the ONL of the dHC retina. Immunohistochemical and electron microscopic analyses showed that synaptic contacts between PRs and BCs are maintained in the dHC retina. Electrophysiological analysis using a multi-electrode array showed that center-surround receptive field formation of RGCs is impaired in the dHC retina, which supports previous studies showing that HCs regulate antagonistic center-surround receptive field organization^4^. In addition, we found the reduction of both the ON and OFF response diversities in RGCs, impairment of global light adaptation at the retinal output level, and alteration of spatial frequency tuning in the dHC retina. We also found that the optimal spatial frequency of OKRs optokinetic responses (OKRs) decreased in the dHC mice than that in the control mice. Our results suggest that HCs are involved in multiple mechanisms of retinal information processing.

## Results

### Generation of retinal horizontal cell-depleted (dHC) mice

**Figure 1:**
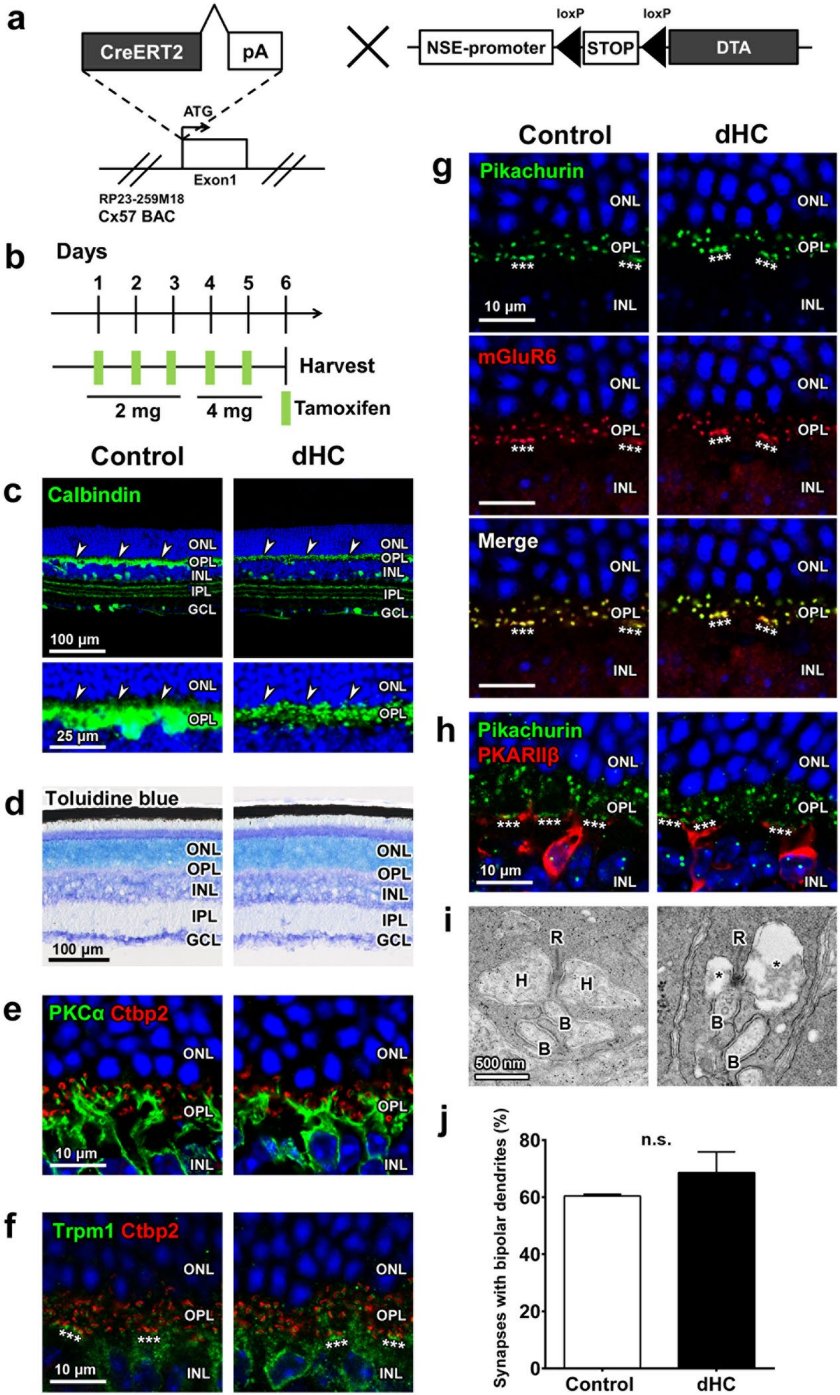
Histological characterization of the dHC retina. (**a**) Schematic diagram of the *BAC-Cx57-CreERT2* construct, in which the *CreERT2-pA* cassette is integrated into the translation start site of the mouse *Connexin57* (*Cx57*) gene, and the *NSE-DTA* construct, in which the expression of Cre-mediated diphtheria toxin A (DTA) is induced under the control of *neuron-specific enolase* (*NSE*) promoter. (**b**) Schematic diagram of schedule for tamoxifen administration and harvest of retinas. (**c**) HCs were immunostained with an anti-Calbindin antibody (arrowheads) in control and dHC retinas. (**d**) Toluidine blue staining of control and dHC retinas. (**e**–**h**) Immunohistochemical analysis of synaptic connections between PRs and BCs in control and dHC retinas using marker antibodies as follows: PKCα (rod BC), Ctbp2 (synaptic ribbon), Trpm1 (ON BC), Pikachurin (PR terminal), mGluR6 (BC dendritic terminal), and PKARIIβ (type 3b OFF cone BC). Asterisks indicate cone terminals. (**i**) Ultrastructural analysis of PR ribbon synapses in control and dHC retinas by transmission electron microscopy. B, BC dendrite; H, HC process; R, synaptic ribbon. Asterisks indicate space due to lack of HC dendrites. (**j**) Quantification of PR ribbon synapses containing bipolar cell dendrites. The numbers of ribbon synapses quantified are 134 (rods, 130; cones, 4) for control and 105 (rods, 103; cones, 2) for dHC retinas (n = 3 retinas from three animals for each genotype). Nuclei were stained with DAPI (blue). ONL, outer nuclear layer; OPL, outer plexiform layer; INL, inner nuclear layer; IPL, inner plexiform layer; GCL, ganglion cell layer. Data are presented as mean ± SD. *p* = 0.12. n.s., not significant.

The promoter activity of the *Connexin57* (*Cx57*) gene is known to be active only in retinal HCs and the thymus medulla^27, 28^. To establish the dHC mouse line, we generated BAC-Cx57-CreERT2 transgenic mice (Fig. 1a). We first crossed BAC-Cx57-CreERT2 mice with a reporter mouse line, R26-CAG-loxP-mTFP1 (Supplementary Fig. 1a)^29^. We injected tamoxifen into these mice at 2 to 3 months of age and found that mTFP1 signals were restricted to the OPL in the retina and overlapped those of Calbindin (arrowheads) (Fig. 1b and Supplementary Fig. 1b,c). This observation shows that HC-specific Cre recombinase activity can be induced by tamoxifen injection in the BAC-Cx57-CreERT2 retina.

We next mated BAC-Cx57-CreERT2 mice with NSE-DTA mice, in which the expression of Cre-mediated diphtheria toxin A (DTA) is induced under the control of the *neuron-specific enolase* (*NSE*) promoter^21^ (Fig. 1a). Since one molecule of DTA introduced into a cell is sufficient to kill the cell^30^, HC death is expected to be immediate after tamoxifen injection. To examine the long-term effect of HC loss, we performed histological analysis of the retina from dHC mice at 2 weeks after the last tamoxifen injection (Supplementary Fig. 2a). Age- and genetic background-matched NSE-DTA mice without the BAC-Cx57-CreERT2 allele were treated with tamoxifen and used as control mice. HCs were totally fragmented in the dHC retina (Supplementary Fig. 2b, arrowheads), whereas the number and morphology of Calbindin^+^ cells in the inner retina were unaltered (Supplementary Fig. 2b). Consistent with a previous report^26^, we observed that aberrantly sprouting dendrites of BCs make ectopic synaptic contacts with PR termini (Supplementary Fig. 2c).

### Synaptic contacts between photoreceptor and bipolar cells are maintained shortly after horizontal cell depletion

Since long-term removal of retinal HCs may disturb normal synaptic connection between PRs and BCs, we prepared retinal sections from dHC mice at 1 day after the last tamoxifen injection (Fig. 1b). As observed in the 2-week condition (Supplementary Fig. 2a), HCs were totally fragmented in the dHC retina, whereas the number and morphology of Calbindin^+^ cells in the inner retina were comparable between control and dHC mice (Fig. 1c and Supplementary Fig. 3a). There was no significant difference in the thickness of the total retinal layer or the ONL between control and dHC mice in the 1-day condition (Fig. 1b,d and Supplementary Fig. 3b,c). We then immunostained major cell types in the control and dHC retinas, and found that the number of rod, cone, bipolar, amacrine, ganglion, and Müller glial cells are unchanged between control and dHC retinas (Supplementary Fig. 4a–e). In both control and dHC retinas, ON BC dendritic tips were located in the vicinity of PR ribbons (Fig. 1e,f). Pikachurin (a photoreceptor terminal marker) signals overlapped mGluR6 (an ON BC dendritic tip marker) signals (Fig. 1g), and cone PR terminals labeled with Pikachurin clusters were adjacent to cone OFF BC dendrites (Fig. 1h). We did not observe ectopic synapse formation in the ONL of either control or dHC retinas (Fig. 1e–h). In the control retina, typical ribbon synapses containing a single ribbon with HC processes and BC dendrites were observed (Fig. 1i). Although PR ribbon synapses in dHC mice contain cell debris due to HC death, single ribbons with BC processes were observed in the dHC retina (Fig. 1i). We immunostained the retinas from the same mice used for electron microscopic analysis with an anti-Calbindin antibody, and confirmed that HCs are totally fragmented in the dHC retina. There was no significant difference in the number of PR ribbon synapses containing BC dendrites between control and dHC retinas (Fig. 1j). These results suggest that synaptic connections between PRs and BCs are unaltered shortly after the depletion of HCs. From these observations, we selected the 1-day condition to perform the following experiments (Fig. 1b).

### Horizontal cell depletion influences the firing properties of RGCs

**Figure 2:**
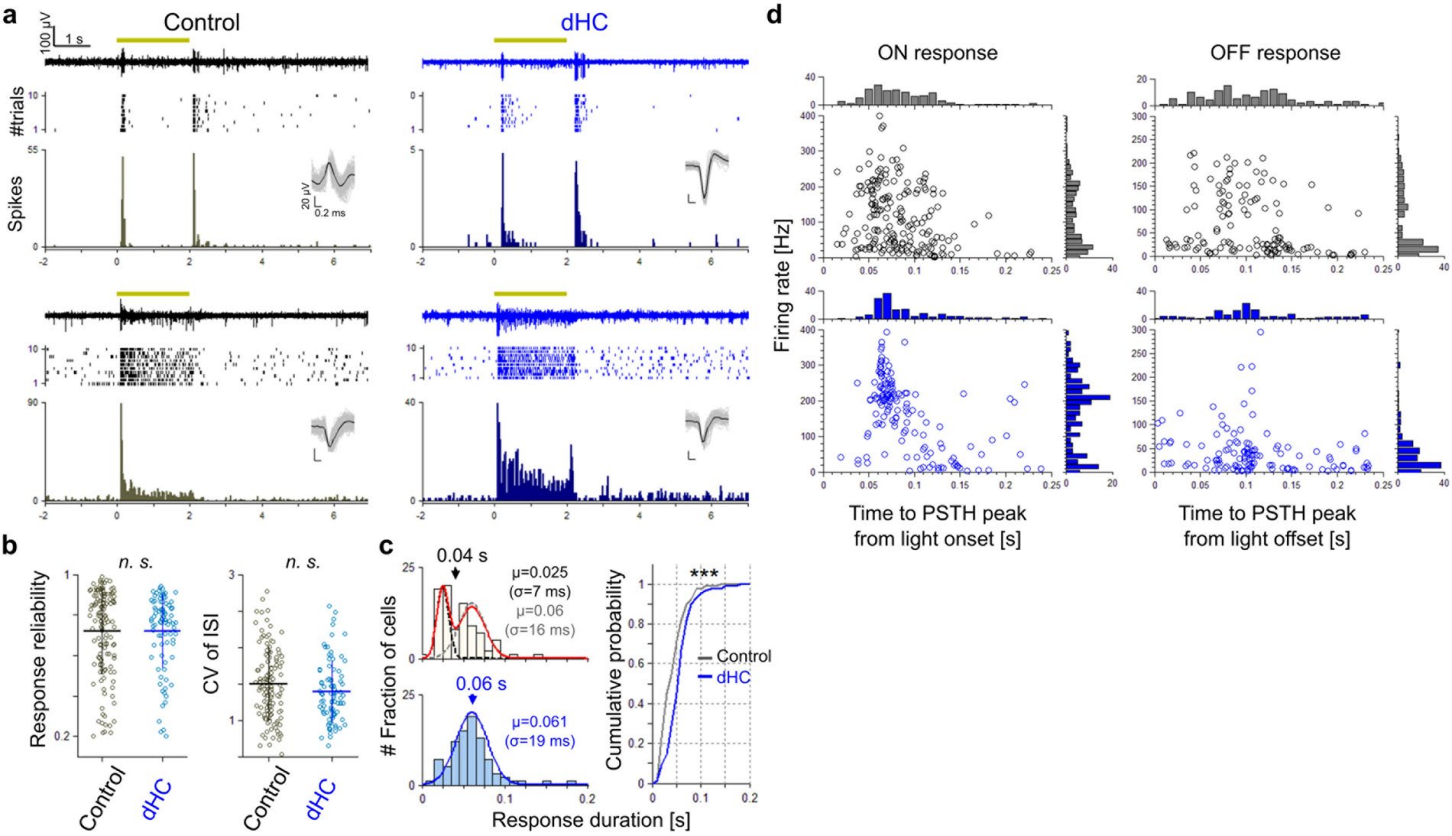
Changes in firing properties of RGCs by the depletion of horizontal cells. (**a**) Light-evoked responses recorded from control and dHC retinas. Examples of transient (upper) and sustained (lower) responses evoked by light stimulation (1,600 × 1,600 μm square, 2 s, yellow). Raw data for one trial (top), spike raster plots (middle), and the PSTH (30 ms bin, 10 trials, bottom). Inset, sorted spikes. (**b**) Left, Pearson’s *r* between trials. Right, CV of inter-spike interval. These measures were calculated from light onset (0 s) to 4 s. Gray, 186 RGCs in 6 control retinas; cyan, 136 RGCs in 4 dHC retinas. Bars, mean ± SD; n.s., not significant. (**c**) Left, histograms of response duration (upper, control; lower, dHC). Duration is defined as a half width at half maximum of PSTH. Histograms are fitted by Gaussian functions (control, red line, sum of two Gaussians, each of which correspond to transient [dotted black] and sustained [dotted gray]; dHC, blue line, single Gaussian). Right, cumulative probability of the histograms. gray, control. blue, dHC. *p* = 5.05 × 10^−4^. (**d**) Population analysis of light-evoked responses. Abscissa, time to the PSTH peak from the light onset or from the light offset. Ordinate, median firing rate for each RGC. Distribution of light-evoked RGC response property in the dHC retinas was different from that in control retinas (114 ON response and 108 OFF response in control retinas, 78 ON response and 79 OFF response in dHC retinas; ON latency, Kolmogorov-Smirnov test, *p* = 0.0044, OFF latency, Kolmogorov-Smirnov test, *p* = 6.59 × 10^−6^; ON firing rate, Kolmogorov-Smirnov test, *p* = 6.58 × 10^−6^, OFF firing rate, Kolmogorov-Smirnov test, *p* = 7.77 × 10^−11^).

To investigate the influence of HC depletion on retinal encoding, we recorded the firings of RGCs by applying a multi-electrode array to the isolated retina. Figure 2a shows light-evoked responses of four example RGCs. We examined the firing properties based on several measures (Fig. 2b and Supplementary Fig. 5)^31, 32^. The response reliability of the dHC retina was not different from that of the control retina (Fig. 2b *left*, Pearson’s correlation between trials, unpaired *t*-test, *p* = 0.58; Supplementary Fig. 5a, Fano factor, unpaired *t*-test, *p* = 0.68). In addition, the temporal characteristics of light-evoked firing were not different between dHC and control retinas (Fig. 2b *right*, CV of inter-spike interval, unpaired *t*-test, *p* = 0.33), suggesting that light signal transmission from PRs to RGCs is maintained in the dHC retina. We also examined the influence of HC depletion on the direction selective circuit^33, 34^. We found that the proportion of DS RGCs to total RGCs in the dHC retina was unchanged from that in the control retina (Supplementary Fig. 6, ON-DS RGCs, 18% in control, 16% in dHC; ONOFF-DS RGCs, 23% in control, 24% in dHC, Chi-squared test, *p* = 0.81). Furthermore, the tuning of directional selectivity and preferred direction of DS RGCs were similar in both dHC and control retinas (Supplementary Fig. 7, median of distribution of the direction selectivity index: ON-DS RGCs, 0.49 in control, 0.48 in dHC, Kolmogorov-Smirnov test, *p* = 0.37, ONOFF-DS RGCs, 0.51 in control, 0.52 in dHC, Kolmogorov-Smirnov test, *p* = 0.16; Supplementary Fig. 8, proportion of preferred direction: ON-DS RGCs, Kolmogorov-Smirnov test, *p* = 0.28, ONOFF-DS RGCs, Kolmogorov-Smirnov test, *p* = 0.09).

In contrast to the persistent generation of the light-evoked firing, the response pattern was influenced (Fig. 2c,d). In the control retina, the response pattern was variable from transient to sustained (Fig. 2a *left*). The response duration histogram showed bimodal distribution (Fig. 2c *left*, upper, red), which could be described by the sum of two Gaussians (mean (s): dotted black, μ = 0.025; dotted gray, μ = 0.06), indicating that RGCs in the control retina are categorized into the transient and sustained types. In the dHC retina, however, most RGCs evoked sustained responses (Fig. 2a *right*). The response duration histogram showed a unimodal distribution (Fig. 2c *left*, lower, blue), which could be described by a single Gaussian (mean (s): blue, μ = 0.061). The cumulative probability function of the response duration revealed significant differences between dHC and control retinas (Fig. 2c *right*, Kolmogorov-Smirnov test, *p* < 0.001). We also recognized that light-evoked responses differ in the time-to-peak latency and median firing rate between the dHC (Fig. 2d, blue) and control retinas (Fig. 2d, black). Responses of RGC population in the dHC retina showed a shorter latency and a higher firing rate than those in the control retina (median of latency distribution (ms): ON, 80 in control, 70 in dHC, OFF, 110 in control, 100 in dHC; median of firing rate distribution (Hz), ON, 90 in control, 190 in dHC, OFF, 25 in control, 35 in dHC, Kolmogorov-Smirnov test, *p*s < 0.001). These results show that the depletion of HCs reduces the diversity of RGC response properties (Fig. 2d, the euclidean distance between the average and each data point: ON, 77.1 in control, 50.8 in dHC; OFF, 58.9 in control, 36.3 in dHC, unpaired *t*-test, *p*s < 0.01), which would be essential for parallel visual information processing in the retina^33^ (see Discussion).

### Horizontal cell depletion influences the receptive field characteristics of RGCs

**Figure 3:**
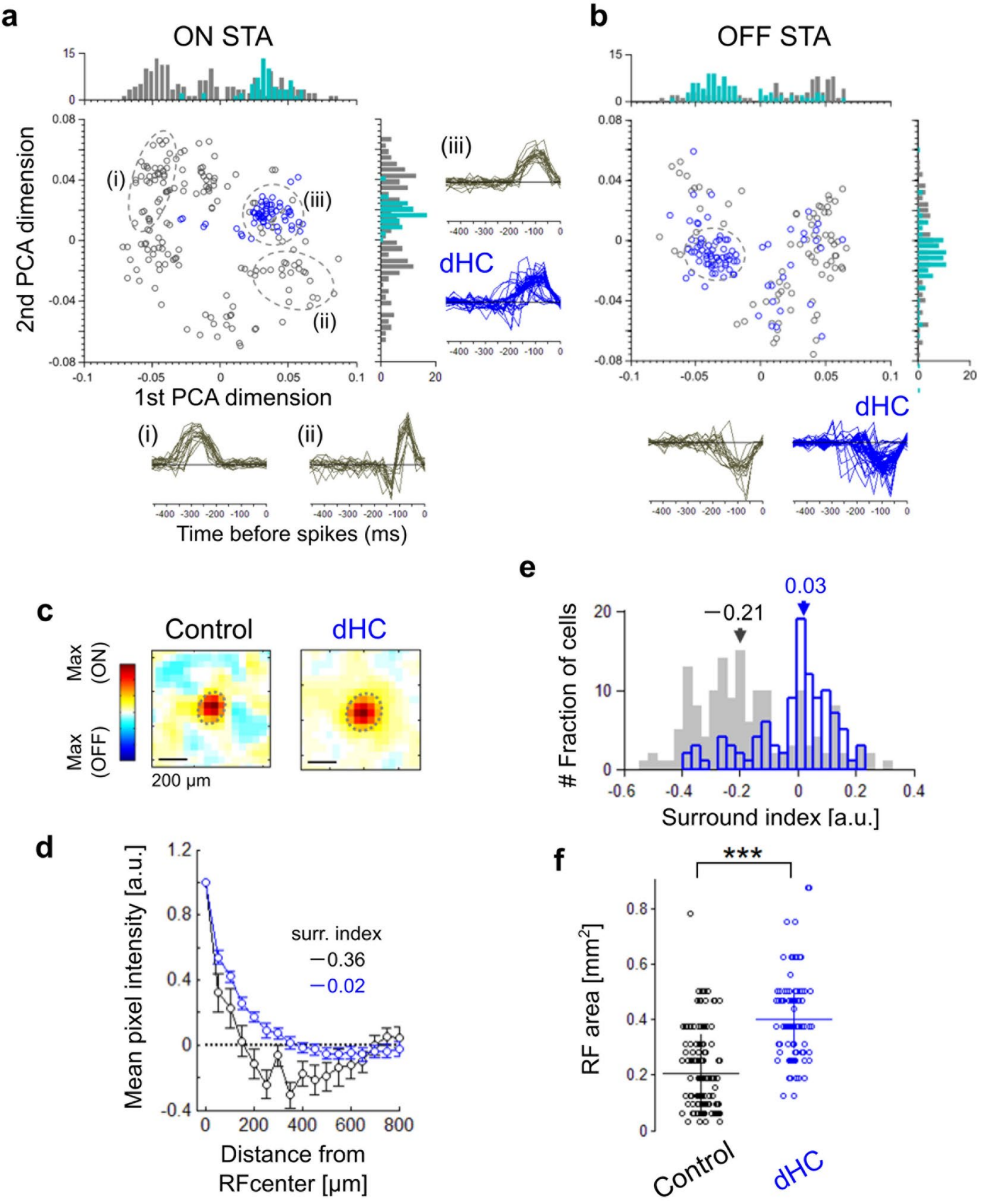
Horizontal cells mediate the surround inhibition of RGCs. (**a**,**b**) Principal component analysis for the aggregate of computed ON STAs (**a**) and OFF STAs (**b**). gray, 148 RGCs in 3 control retinas. blue, 134 RGCs in 3 dHC retinas. Histograms were calculated by 0.004-bin in each axis. insets, examples of STAs. Areas denoted by dotted ines showed aggregation of STAs (gray, control; blue, dHC). In ON STAs, RGCs within the first quadrant, which show the fast time course, were 93% of total 62 RGCs in the dHC retinas (blue, dHC). In OFF STAs, RGCs within the third quadrant, which show the fast time course, were 78% of the total 72 RGCs in the dHC retinas (blue, dHC). (**c**) Examples of the estimated RFs of the control (left) and dHC (right) retinas. (**d**) Relationship between the distance from the RF center and the mean pixel intensity (black, control; blue, dHC) of the sample RFs (**c**). Values were normalized by the mean intensity at the RF center. bars, SD. Inset values, surround indexes denoted minimum mean intensity. (**e**) Histograms of surround indexes calculated by 0.03-bin. inset numbers, median of each distribution (gray, control; blue, dHC). (**f**) Area of the RF estimated for each RGC in control and dHC retinas. bars, mean ± SD, unpaired *t*-test, *p* = 2.21 × 10^−22^, ***p < 0.001.

It is unclear how extensively HCs contribute to the center-surround RF organization of RGCs^2^. We analyzed the spatiotemporal RF properties of RGCs estimated by the reverse correlation method^35, 36^. First, to analyze the temporal profile of the RF, we computed the spike-triggered average (STA) of RGCs and decomposed the population of STAs into two dimensions by principal component analysis (Fig. 3a, ON STA; Fig. 3b, OFF STA). In the control retina, the STA profile plot showed a dispersed distribution of clusters (Fig. 3a,b, gray), indicating the presence of various RGC subtypes^37^ (Fig. 3a, (i)–(iii)). In the dHC retina, however, the STA profile plot showed a limited dense distribution (Fig. 3a,b, blue; SD of distribution: ON STA, 1st PCA axis, 0.04 in control, 0.017 in dHC; 2nd PCA axis, 0.03 in control, 0.007 in dHC; OFF STA, 1st PCA axis, 0.034 in control, 0.027 in dHC; 2nd PCA axis, 0.02 in control, 0.014 in dHC; distribution difference between control and dHC retinas: ON STA, Kolmogorov-Smirnov test: 1st PCA axis, *p* = 7.69 × 10^−16^; 2nd PCA axis, *p* = 6.04 × 10^−8^; OFF STA, Kolmogorov-Smirnov test: 1st PCA axis, *p* = 4.28 × 10^−4^; 2nd PCA axis, *p* = 5.82 × 10^−38^), and most RGCs had a RF temporal profile with fast time-to-peak latency and prolonged integration time (Fig. 3a,b, the time-to-peak latency of STA (ms): ON, −146.09 ± 68.36 in control, −96.87 ± 35.79 in dHC; OFF, −131.05 ± 82.06 in control, −115.84 ± 74.09 in dHC; note that SD of the time-to-peak latency was smaller in RGCs of the dHC retina than those of the control retina). Next, we analyzed the spatial profile of the RF (Fig. 3c–f). In the control retina, some RGCs showed obvious surround inhibition (Fig. 3c *left*). We calculated the mean intensity of pixels as a function of distance (every 50 pixels) from the RF center. The surround index of a RGC was defined as the normalized minimal mean intensity (Fig. 3d,e, see Methods). The surround index was negative in an example of ON RGCs in the control retina, indicating the presence of an antagonistic surround (Fig. 3c *left*, and Fig. 3d, black). However, in an example of ON RGCs in the dHC retina, the surround index was nearly 0, showing negligible antagonistic surround (Fig. 3c *right*, and Fig. 3d blue). The surround index histogram indicates that many RGCs in the dHC retina had poor antagonistic surround (Fig. 3e, median of surround index: −0.21 in control; 0.03 in dHC). Furthermore, the RF center size of RGCs in the dHC retina was significantly larger than that in the control retina (Fig. 3f, RF area (mm^2^): 0.21 ± 0.14 in control; 0.4 ± 0.3 in dHC), indicating the reduction of surround antagonism. These results indicate that the loss of HCs impairs the spatial tuning of RGC activity. It has been reported that inhibitory inputs from ACs in the IPL contribute to formation of the RGC antagonistic surround^34^. However, the present results show that the inhibitory activities of ACs are insufficient to compensate for the center-surround antagonism of RGCs in the dHC retina. Therefore, we suggest that HCs play an important role in formation of the antagonistic RF surround in RGCs.

### Effects of horizontal cell loss on tuning for spatiotemporal frequency of visual motion

**Figure 4:**
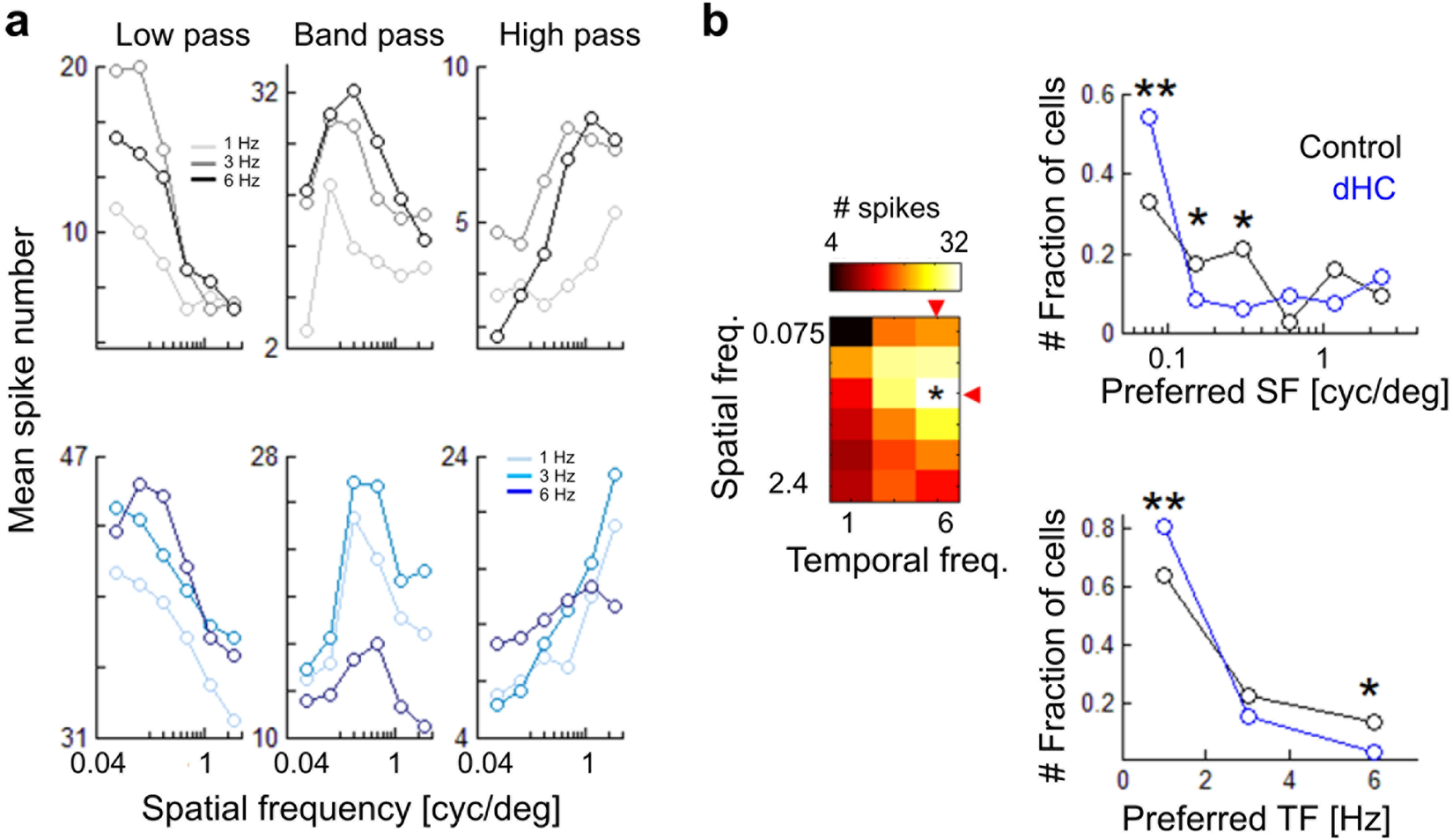
Responses to the drifting gratings. (**a**) Response examples of three types of frequency tuning. Upper, control; lower, dHC. Mean spike numbers were calculated from spikes evoked during drifting phase. (**b**) Left, matrix of observed spike numbers. We quantified the frequency preference of each cell as a frequency condition (red triangles) causing the max response (asterisk). Right, histograms of the frequency preference (black, control; blue, dHC). Upper, SF; lower, TF. Multiple comparisons were based on the adjusted standardized residual (**p < 0.01, *p < 0.05). 159 RGCs in 4 control retinas. 161 RGCs in 4 dHC retinas. *p* = 0.0067 (SF, 0.0075), *p* = 0.0334 (SF, 0.15), *p* = 0.042 (SF, 0.3), *p* = 0.075 (SF, 0.6), *p* = 0.195 (SF, 1.2), *p* = 0.337 (SF, 2.4), *p* = 0.009 (TF, 1), *p* = 0.207 (TF, 3), *p* = 0.015 (TF, 6).

To test how the changes in spatiotemporal properties of RGC RF in the dHC retina influence visual motion encoding, we analyzed the responses to drifting gratings with various spatial and temporal frequencies (SF: 0.075, 0.15, 0.3, 0.6, 1.2, 2.4 cyc/deg; TF: 1, 3, 6 Hz). In both control and dHC retinas, RGCs respond to visual motion (Firing index, (*R*_*drift*._ − *R*_*base*_) / (*R*_*drift*._ + *R*_*base*_), *R*, mean # of spikes: 0.58 ± 0.37 in control, 0.59 ± 0.32 in the dHC, Kolmogorov-Smirnov test, *p* = 0.38, Supplementary Fig. 5b), and show diverse spatial and temporal frequency dependence (Fig. 4a). The most effective spatiotemporal frequency was determined by the firing to drifting gratings for each cell (Fig. 4b *left*, red triangles and asterisk). In the dHC retina, many RGCs responded better to lower spatial frequencies (Fig. 4b *upper right*, preferred SF (cyc/deg), 0.52 ± 0.71 in control, 0.34 ± 0.56 in dHC, Chi-squared test, *p*s < 0.001) and temporal frequencies (Fig. 4b *lower right*, preferred TF (Hz), 2.25 ± 1.84 in control, 1.89 ± 1.38 in dHC, Chi-squared test, *p*s < 0.001) than those in the control retina. These results indicate that ablation of HC activity does not spoil visual motion encoding but rather influences the frequency tuning of RGCs. It is likely that the influence on the frequency tuning may be ascribed to changes in RF and firing properties. Enlargement of the RF size (Fig. 3f) may reduce the spatial resolution. Reduction of the sustained input for firing (i.e., prolonged integration time; Fig. 3a,b) and prolongation of the response duration (Fig. 2c) may decrease the temporal resolution.

### Horizontal cells are essential for retinal global light adaptation

**Figure 5:**
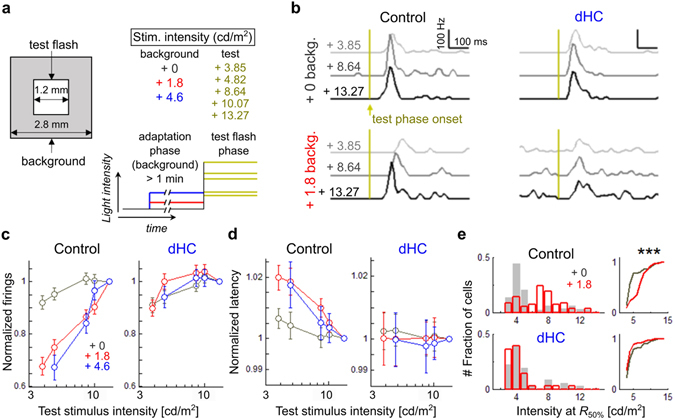
Global light adaptation mediated by horizontal cells. (**a**) Stimulus schema. Intensity of the test flash (1,200 × 1,200 μm square, 2 s) was changed (3.85, 4.82, 8.64, 10.07, 13.27 cd/m^2^) on various background intensities (2,800 × 2,800 μm square, 0, 1.8, 4.6 cd/m^2^). (**b**) Responses to the test flash (3.85, 8.64, 13.27 cd/m^2^, PSTH of 0.01 s-bin) on the background with low (black; 0 cd/m^2^) or medium intensity (red; 1.8 cd/m^2^). yellow, test flash onset. Left, control. Right, dHC. (**c**) Spike number for 0.4 s from the test flash onset on various background intensities. Values were normalized to the spike number during the maximum test flash. Bars, mean ± SD. (**d**) Time-to-peak latency of the PSTH (denoted in **c**) among various background intensities. Values were normalized to the latency during the maximum test flash. Bars, mean ± SD. (**e**) Left, histograms of the intensity causing 50% of the max spike numbers (*R*_50%_) with the low (gray, +0) or medium (red, +1.8) background intensity. Upper, control; lower, dHC. Right, cumulative distribution of *R*_50%_. 156 RGCs in 4 control retinas. 148 RGCs in 4 dHC retinas. Kolmorov-Smirnov test, *p* = 7.79 × 10^−10^ in control retina, *p* = 0.75 in dHC.

Adaptation to the global mean light level may play a key role in the retinal processing of natural vision^38^. It was previously reported that HC feedback plays a role in the light adaptation of cone PRs^39^, however, the effect of HC feedback on light adaptation at the retinal output level is unknown. To investigate this, we recorded the response to a test flash (Fig. 5a, test flash, 3.85 ~ 13.27 cd/m^2^) superimposed on a background with various luminance intensities (Fig. 5a, background, +0, +1.8, +4.6 cd/m^2^). In the control retina, as the light intensity of the test flash increased from the lowest background intensity (+0 cd/m^2^), the firing rate increased and then saturated (Fig. 5b *upper left* and Fig. 5c gray). To quantify the sensitivity to the test flash at various background intensities, we plotted the firing rate against the test flash intensity (Fig. 5c, note that the firing rate was normalized to the value evoked by the strongest test flash, 13.27 cd/m^2^). The response at low background saturated at 8.64 cd/m^2^ (Fig. 5c gray, paired *t*-test with Bonferroni correction, 8.64 cd/m^2^ vs 10.07 cd/m^2^, *p* = 0.78). At the medium background intensity (8.64 cd/m^2^), the firing rate of responses to the test flash, which evoked saturated firings at the low background intensity (Fig. 5b *bottom left*), increased further, indicating that the dynamic range of responses shifted toward higher intensity (Fig. 5c red, paired *t*-test with Bonferroni correction, 8.64 cd/m^2^ vs 10.07 cd/m^2^, *p* = 0.026; note that the slope before saturation was almost linear). In addition, the time-to-peak latency became shorter as the test flash intensity was increased, showing another aspect of light adaptation (Fig. 5b,d; note that the slope of the latency function became steeper as the background intensity increased). On the other hand, in the dHC retina, as the flash intensity increased at the medium or high background intensity (Fig. 5c,d red and blue), neither the firing rate nor the time-to-peak latency changed obviously (Fig. 5b–d). Population analysis revealed that the test flash intensity which evoked the half-maximum response (*R*_50%_) shifted toward higher flash intensities at the medium background intensity in RGCs of the control retina (Fig. 5e *upper*, median intensity at *R*_50%_ (cd/m^2^), 3.86 at the low background intensity, 5.31 at the medium background intensity, Kolmogorov-Smirnov test, *p* < 0.001) but not in RGCs of the dHC retina (Fig. 5e *bottom*, median intensity at *R*_50%_ (cd/m^2^), 3.76 in the low background intensity, 3.28 at the medium background intensity, *p* = 0.75, *n.s*.). These results suggest that HCs play an essential role in global light adaptation to the surrounding environment.

### Visual function of dHC mice is altered in OKRs

**Figure 6:**
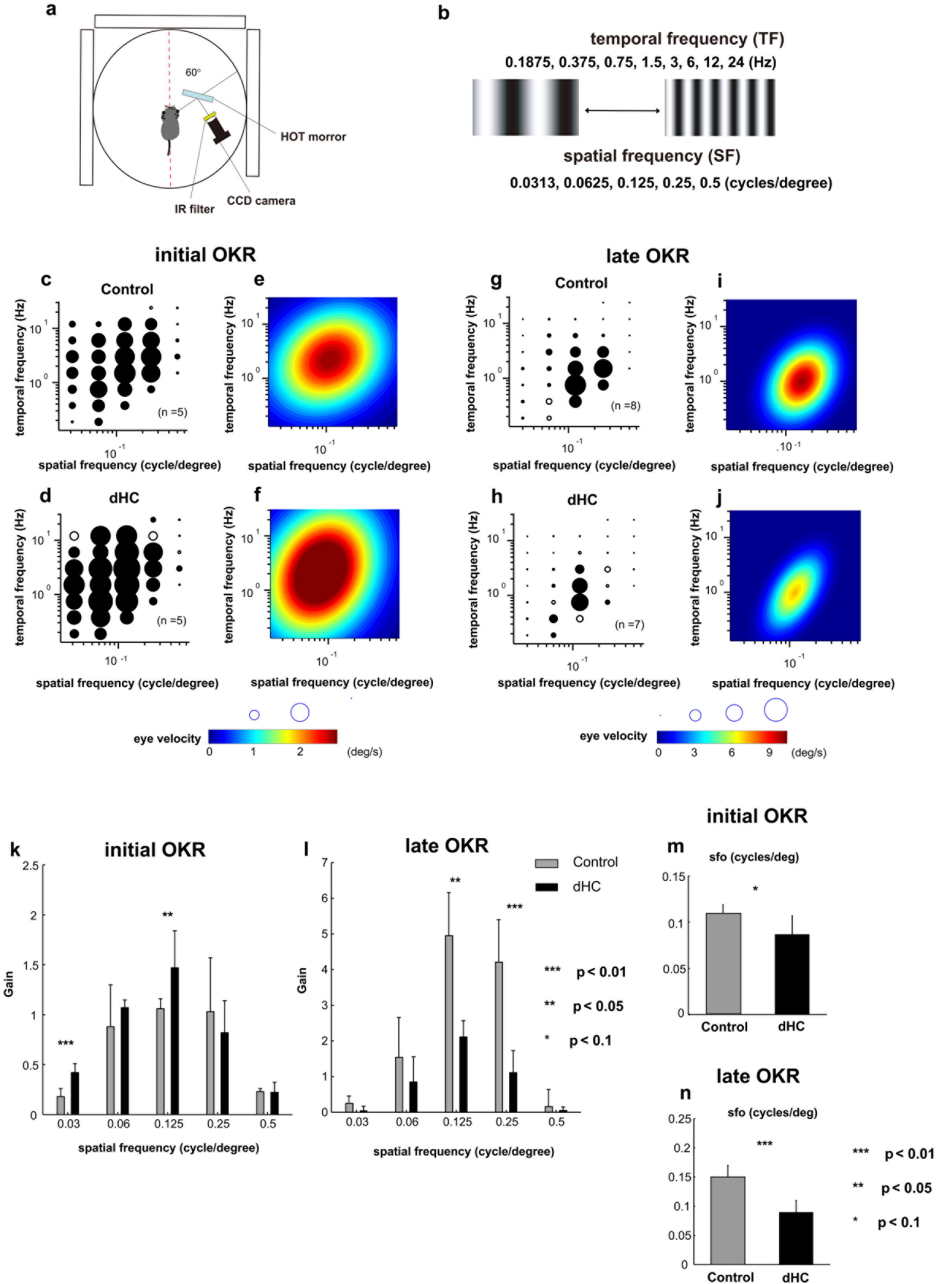
Optokinetic responses (OKRs) in dHC mice. (**a**) The visual stimulus presented on three LCD monitors set around the mouse (19-inch LCD; refresh rate, 75 Hz; size, 270° × 65.7°). (**b**) The visual stimuli were moving sinusoidal gratings with one of five spatial frequencies (SF) selected randomly from lookup table: 0.0313, 0.0625, 0.125, 0.25, or 0.5 cycles/deg in a given trial. The temporal frequency (TF) was selected from 0.1875, 0.375, 0.75, 1.5, 3, 6, 12, or 24 Hz. (**c**–**f**) Eye velocities of the initial OKR responses averaged over the mice are shown by diameter of the circle. Filled circles: statistically significant (*t*-test, *p* < 0.05) responses (**c,d**). Heat map plots of the best-fit Gaussian functions (**e,f**). (**c,e**: control mice (n = 5), **d,f**: dHC mice (n = 5)). (**g**–**j**) Mean amplitudes of the slow phase eye velocity of the late OKR response represented by the diameter of the circle. Filled symbols: statistically significant (*t*-test, *p* < 0.05) responses (**g,h**). Heat map plots of the best-fit Gaussian functions (**i,j**). (**g,i**: control mice (n = 8), **h,j**: dHC mice (n = 7)). (**k,l**) Comparison of properties of the spatial frequency in initial and late OKRs. The gain (eye velocity/ stimulus velocity) of the eye movement was calculated. The sum of gains at all temporal frequencies at each spatial frequency is shown. (**k**: initial OKR, control mice (n = 5), dHC mice (n = 5), *p* = 0.00322 (SF 0.03), *p* = 0.3673 (SF 0.06), *p* = 0.0497 (SF 0.125), *p* = 0.475 (SF 0.25), *p* = 0.943 (SF 0.5), **l**: late OKR, control mice (n = 8), dHC mice (n = 7), *p* = 0.185 (SF 0.03), *p* = 0.448 (SF 0.06), *p* = 0.0450 (SF 0.125), *p* = 0.0088 (SF 0.25), *p* = 0.657 (SF 0.5)). (**m,n**) The difference in the optimal spatial frequency (sfo) (**m**: initial OKR, **n**: late OKR). *p* = 0.091 (**m**), *p* = 0.0028 (**n**). Data are presented as mean ± SD.

To investigate the effect of HC depletion on visual function, we examined OKRs of the control and dHC mice (Fig. 6a–n). OKRs are reflexive eye movements elicited by a moving visual pattern^40, 41^ (Fig. 6a,b). The initial phase of OKRs is observed in a very short period within 500 ms after visually sensing a moving object, while the late phase of the OKRs (also known as optokinetic nystagmus: OKN) is a series of eye movements alternating slow tracking and quick resetting to initial eye position within a longer time period (30 sec in this study).

We quantitatively measured the OKRs in control and dHC mice by plotting mean eye velocities for each visual stimulus in a coordinate system of spatial and temporal frequencies (Fig. 6c,d; initial phase, Fig. 6g,h; late phase). We analyzed the slow phase eye velocity of OKNs as the late phase of OKRs. To characterize the optimal spatiotemporal frequencies, we also visualized the quantified OKR responses of control and dHC mice as heat-maps (Fig. 6e,f; initial phase, Fig. 6i,j; late phase). In the initial phase of the OKR, the dHC mice showed larger initial responses to the optokinetic stimuli with low spatial frequencies compared with those of the control mice (Fig. 6c–f). On the other hand, in the late phase of the OKR, the responses of the dHC mice were somewhat weaker than those of the control mice (Fig. 6g–j). To evaluate the OKRs, we quantified the eye velocities of the control and dHC mice at each spatial frequency tested. In the initial OKR, the gain of the dHC mice was larger than that of the control mice at relatively low and middle frequencies, but was unchanged at relatively high frequencies (Fig. 6k). On the other hand, the gain of the late OKRs of the dHC mice was smaller than that of the control mice at the middle frequencies, but was unaltered at low and high frequencies (Fig. 6l). The changes of the initial OKRs might be explained by the changes in activity of slow-motion-tuned DS RGC^42^. We found that the response latency of slow-motion-tuned DS RGC in the dHC retina was shorter than that in the control retina (Supplementary Fig. 9; mean response latency (in ms) of slow-motion-tuned ON-DS was 77.19 ± 18.02 in control and 55.31 ± 13.38 in the dHC, unpaired *t*-test, *p* < 0.001; and slow-motion-tuned ONOFF-DS was 87.76 ± 29.78 in control and 67.47 ± 17.4 in the dHC, unpaired *t*-test, *p* < 0.05). A faster response in slow-motion-tuned ON DS RGC might increase the velocity of the initial OKR (see Discussion).

We did not observe any significant differences in temporal frequency properties of both initial and late phase OKRs between the control and dHC mice (Fig. 6c–j). Alterations in temporal frequency tuning of RGCs due to HC loss (Fig. 4b) may not be sufficient to affect the temporal frequency properties of OKRs. In contrast, the optimal spatial frequencies of both the initial and late phase of the dHC mice were significantly lower than those of the control mice (Fig. 6c–j,m,n), which is consistent with the observations in RGCs (Fig. 4b). These results suggest that HCs regulate spatial frequency properties not only in RGCs but also at an individual level.

## Discussion

### The dHC retina exhibited impaired synaptic gain control and light adaptation

In the current study, we examined HC function using dHC mice. In immunohistochemical analysis we observed that all of the HCs became fragmented but no obvious change was detected in other parts of the dHC retina (Fig. 1c–h and Supplementary Figs 3 and 4). Electron microscopic observation showed that synaptic contacts between PRs and ON BCs are maintained in the dHC retina (Fig. 1i,j). In both dHC and control retinas, RGCs responded to light stimulation with similar reliability (Fig. 2a,b and Supplementary Fig. 5), suggesting that HC loss does not spoil light signal transmission from PRs to RGCs. Although we cannot completely exclude the possibility of secondary effects resulting from HC loss, animal models depleted in a certain cell type have been used to understand the *in vivo* function of the targeted cell type in the retina^18–20^. In a previous study, retinal electrophysiological and behavioral properties were analyzed in mice manifesting rod degeneration and ectopic synapse formation in the ONL as a consequence of HC depletion in the adult stage^26^. In contrast, in this study, we performed multi-electrode array and OKR analyses using dHC mice, in which those histological changes in the retina were not detected (Fig. 1c–h and Supplementary Figs 3 and 4).

Depletion of HCs changed the firing properties of RGCs (Fig. 2c,d). Prolonged light responses with short latencies and high firing rates indicate that the synaptic gain control mechanisms in the OPL are impaired in the dHC retina. HCs modify the synaptic transmission from PRs to BCs and prevent the saturation of neural activity in the OPL. Electrophysiological and pharmacological studies showed that the ephaptic mechanism mediates the feedback control of the synapses in the OPL^8, 10, 11, 43^. Hemichannels expressed in HC dendrites may play an important role in modulating voltage-dependent Ca^2+^ channels in the PR terminal^17, 44^. Recent works showed that HC feedback to PR terminals mediated by H^+^ released from HCs regulates the activity of voltage-dependent Ca^2+^ channels^14, 16, 45^. In the dark, glutamate release from PRs is downregulated by the acidification of Ca^2+^ channels in the PR terminal mediated by H^+^ released from HCs, whereas light-evoked hyperpolarization of HCs causes a decrease of H^+^ and alkalinization of the Ca^2+^ channels, resulting in upregulation of the glutamate release from PRs. GABA release through GABA autoreceptors on HCs may also modulate the hemichannels on the HC dendrites, while the ephaptic mechanism may regulate glutamate release from cone PRs^17^.

Thus it is likely that loss of feedback from HCs to PRs causes an increase of the glutamate release from PRs to ON BCs in the dark-adapted condition, resulting in hyperpolarization of ON BCs. Hyperpolarization may de-inactivate the T-type Ca^2+^ channels expressed in the ON BC terminals of mice^46^ and enhance the glutamate release from ON BCs, which results in fast and strong responses in RGCs to weak light stimulation. The downregulation of the glutamate release from ON BCs in the dark may also de-inactivate the ionotropic glutamate receptors expressed in RGCs. In the OFF pathway, it is also likely that OFF BCs receive excess excitatory inputs from PRs in the dark due to the loss of HC activity. In addition, it is possible that AII ACs in the dHC retina receive enhanced excitatory inputs from ON BCs (e.g. rod bipolar cell)^33^, which are activated excessively by the loss of HC feedback during light stimulation. The strong inhibitory activity of AII ACs may hyperpolarize OFF BCs and de-inactivate their Ca^2+^ channels. Thus the enhanced glutamate release from OFF BCs may result in fast firing of RGCs at the light offset. Such hypersensitization to light stimulation in the dHC retina may saturate the response to weak light stimulation and reduce the dynamic range of responses in RGCs (Fig. 5). On the other hand, in the control retina, under dark-adapted conditions, HCs control the synaptic gain in the OPL and, in turn, the sensitivity of BCs. The control system also works under light-adapted conditions, and prevents the retinal cells from response saturation. Inhibitory mechanisms mediated by ACs also control the synaptic gain in the IPL^47, 48^. However, reduction of the RF antagonistic surround of RGCs in the dHC retina (Fig. 3e) suggests that the inhibitory mechanism in the IPL is not strong enough to compensate for the loss of HCs in the OPL.

It has been reported that both intracellular Ca^2+^ in the outer segment of PRs^49, 50^ and local contrast adaptation mechanisms in the IPL^48, 51, 52^ contribute to light adaptation. In addition to the local adaptation, adaptation to the global luminance intensity is essential for animals to recognize objects in natural vision. The wide spatial summation area of electrically-coupled HCs^38, 53^ may be suitable for the mechanism of global light adaptation.

It was reported that HCs mediate the cone-driven lateral interaction pathway in the photopic condition. Hyperpolarization of cones, in response to photopic light stimulation, suppresses the activity of HCs, resulting in enhancement of the activity of rods, which in turn drives the rod pathway^54^. If depletion of HCs impairs the HC-mediated rod pathway, it is possible that the deterioration of divergent cone signals change the activity of BCs, resulting in changes in the firing activity of RGCs. In the present study, the light levels of the background ranged from scotopic to mesopic levels and those of the test flash ranged from mesopic to photopic levels^55^. Thus, it is possible that the strong test flash drives the HC-mediated surround inhibition in the control retina. However, deterioration of the HC-mediated surround inhibition in the dHC retina may saturate the firing activity of RGCs at the photopic level.

### Horizontal cells play essential roles in encoding parallel visual information

We observed that the diversity of spatiotemporal frequency channels is impaired in the dHC retina. Diversity of frequency channels is crucial for the parallel encoding of visual information^1, 2, 34^. It is highly likely that HC activity also contributes to parallel visual information transmission to the brain. Ablation of HCs changed the RF properties of RGCs (Fig. 3c–f). In the dHC retina, RGCs showed expanded RF centers (Fig. 3f) with weak antagonistic surrounds (Fig. 3e). The lower shift of optimal spatial frequency in RGCs and OKRs in dHC mice (Figs 4b and 6m,n) may reflect these changes in spatial properties (Fig. 3e,f). Assuming that the receptive field of the dHC RGC is 200~300 μm in diameter (Fig. 3c; 6.7 ~ 10 degrees: note that 1 degree of the visual angle corresponds to ~30 μm in the mouse retina^56^), a grating of 200 ~ 300 μm/cycle (i.e., 0.1 ~ 0.15 cycle/degree) may evoke a strong response because almost the full region of the receptive field along with the weak antagonisitic surround is covered by a stripe of the grating (Fig. 4b). Thus this calculation may explain the preference for lower spatial frequencies in RGCs and OKRs in the dHC mice compared to those in the control mice. In addition, the requirement of the sustained input for firings (Fig. 3a,b) and prolonged firings of RGCs (Fig. 2c) in the dHC retina may decrease the sensitivity to high temporal frequencies (Fig. 4b).

Furthermore, HC depletion reduced the diversity in the RF temporal profile of RGCs (STA, Fig. 3a,b). It has been shown that there are many physiologically distinct subtypes of RGCs in the mouse retina^33, 34, 57^. In the present study, the scatter plot of the light-evoked responses (Fig. 2d) and the decomposed STA characteristics (Fig. 3a,b) show multiple clusters, each of which may correspond to heterogenous RGC subtypes in the control retina. However, RGCs in the dHC retina did not show such diversity. These results suggest that gain control by HCs in the OPL mediates provision of the RGC response diversity. Loss of gain control in the OPL may cause saturation of synaptic transmission in the OPL, which in turn impairs the establishment of a variety of spatiotemporal characteristics in BC responses. The saturated activity of BCs may also cause saturation of AC activity in the IPL. It is possible that the loss of divergent cone signals in the dHC retina mediated by HCs^54^ also influences the activity of bipolar cells. The response diversity of RGCs is determined not only by local circuits in the IPL but also by a global circuit mediated by the lateral interaction of HCs in the OPL.

The initial OKRs were significantly larger in the dHC mouse compared to those in the control mouse (Fig. 6c–f,k). Light-evoked responses of RGCs in the dHC retina showed short latency and high firing rate (Fig. 2d). This may explain the larger responses observed in initial OKRs of the dHC mouse. In addition, it is possible that the response properties of the slow-motion-tuned direction selective ganglion cells (slow-DS RGCs, Supplementary Fig. 6b,c) are changed in the dHC retina. It was reported that most slow ON-DS RGCs project to the accessory optic system^58^, which contributes to the control of OKR initiation^18, 42^. In the dHC retina, the response latency of slow ON- and ONOFF-DS RGCs was shorter than that in the control retina (Supplementary Fig. 9). The faster response of slow-DS RGCs may explain the larger response in initial OKRs of the dHC mouse.

In contrast to the initial OKRs, the late OKRs were significantly smaller in the dHC mouse compared to those in the control mouse (Fig. 6g–j,l). Defects of light adaptation and/or RGC resoponse diversity by loss of HCs could account for this difference in the OKRs between the initial phase and late phase. We observed that adjustment of the sensitivity to ambient light is impaired in the RGCs of the dHC retina (Fig. 5). A longer visual stimulation time may saturate the light response of RGCs, resulting in the reduction of the late OKRs. On the other hand, we found that the diversity of RGCs is reduced in the dHC retina (Figs 2d and 3a,b). Since late OKRs are based on more complicated neural circuits than the initial OKRs, the impairment of RGC response diversity may weaken the late OKRs as compared with the initial OKRs.

Taken together, our results imply versatile functional roles of HCs in the retinal circuit. Previous pharmacological studies showed that HCs regulate antagonistic center-surround RF organization^4^. Our current study using the dHC mouse well supports those findings. In addition, we suggest that HCs play critical roles in global light adaptation at the retinal output level, response diversity of RGCs, and spatial frequency tuning. Our results may provide a new insight into retinal interneuron functions. Understanding detailed molecular mechanisms underlying these HC functions in the retinal circuit and visual function awaits future analyses.

## Methods

### Animal care

All procedures conformed to the ARVO Statement for the Use of Animals in Ophthalmic and Vision Research, and these procedures were approved by the Institutional Safety Committee on Recombinant DNA Experiments (approval ID 3380-6) and Animal Experimental Committees of Institute for Protein Research (approval ID 24-05-1), Osaka University, and animal experiment committees of The University of Tokyo (approval ID 14-14) and Nippon medical school (approval ID H24-4), and were performed in compliance with the institutional guidelines. Mice were housed in a temperature-controlled room at 22 °C with a 12 h light/dark cycle. Fresh water and rodent diet were available at all times.

### Construction of the *BAC-Cx57-CreERT2* transgene

A BAC clone (clone ID: RP23-259M18) containing the entire *Connexin57* (*Cx57*) gene was purchased from the BACPAC Resource Center at the Children’s Hospital Oakland Research Institute (Oakland, CA, USA). Two homologous arms (5′ arm, 912 bp; 3′ arm, 940 bp) from the *Cx57* gene were amplified by PCR using the BAC clone and cloned into pCR-Blunt II-TOPO vector (Invitrogen). Primer sequences are as follows: for 5′ homology arm, forward (F), 5′-GGCGCGCCTAGCACTTGGGAGTCAGAGGCAGGCA-3′ (*Cx57-BoxA-51*), and reverse (R), 5′-CCCGGGGGTTAGGAAGTCACATCTGAAACA-3′ (*Sma-Cx57-BAC rv*); 3′ homology arm, F, 5′-TTAATTAAATGGGAGATTGGAATTTACTGGGT-3′ (*Pac-Cx57-BAC fw*), and R, 5′-GGCCGGCCTACCTCAAGTTGCCTGGGGTGTGGA-3′ (*Cx57-BoxB-31*). Two homologous arms were inserted into both sides of the *CreERT2-pA* cassette in the modified pLD53-SCA-E-B shuttle vector. The *CreERT2-pA* cassette was introduced into the translation start site of the *Cx57* gene by homologous recombination^59^. The correct insertion of the *CreERT2* gene into the *Cx57* locus was confirmed by sequencing.

### Generation of BAC-Cx57-CreERT2 transgenic mice

The entire *BAC-Cx57-CreERT2* transgene was purified as described previously^60^. This purified construct was injected into the pronuclei of fertilized one-cell eggs of B6C3F1 mice. To observe Cre recombinase activities induced by tamoxifen injection in the BAC-Cx57-CreERT2 transgenic mice, we crossed them with R26-CAG-LoxP-mTFP1 reporter strain (#RBRC05147, RIKEN BRC)^29^. BAC-Cx57-CreERT2: R26-CAG-LoxP-mTFP1 mice were then treated with tamoxifen to analyze mTFP1 expression.

### Generation of retinal horizontal cell-depleted (dHC) mice

We mated the BAC-Cx57-CreERT2 transgenic mouse line with the NSE-DTA mouse line^21^. Tamoxifen was injected into the BAC-Cx57-CreERT2: NSE-DTA mouse to deplete retinal HCs. Age- and genetic background-matched NSE-DTA mice without the BAC-Cx57-CreERT2 allele were treated with tamoxifen and used as control mice.

### Tamoxifen treatment

Tamoxifen (Sigma) was dissolved in sunflower seed oil (Sigma) to a concentration of 20 mg/ml and injected intraperitoneally into mice for 5 days. Adult (2- to 3-month-old) mice received 2 mg of tamoxifen on days 1–3 and 4 mg of tamoxifen on days 4 and 5. Animals were used for experiments at 1 day or 2 weeks after the last tamoxifen injection.

### Antibodies

We used the following primary antibodies for immunostaining: mouse monoclonal anti-Brn3a (Chemicon, MAB1585, 1:100), anti-Pax6 (DSHB, 1:100), anti-Ctbp2 (BD Biosciences, 612044, 1:500), anti-GFAP (Sigma, G3893, 1:400), anti-PKARIIβ (BD Biosciences, 610625, 1:500), and anti-RomI (1:500) (a gift from Dr. R. Molday, the University of British Columbia, Canada); rabbit polyclonal anti-Rhodopsin (LSL, LB-5597, 1:2500), anti-M-opsin (Millipore, AB5405, 1:500), anti-Chx10 (1:200)^61^, anti-Pikachurin (1:500)^62^, anti-PKCα (Sigma, P4334, 1:500), anti-Trpm1 (1:100)^63^, and anti-Calbindin (Calbiochem, PC253L, 1:1,000); goat polyclonal anti-S-opsin (Santa Cruz, sc-14363, 1:500) and anti-Brn3b (Santa Cruz, sc-6026, 1:100); guinea pig polyclonal anti-mGluR6 (1:500)^63^; rat monoclonal anti-GFP (Nacalai, 04404-84, 1:1,000) antibodies. We used Cy3-conjugated secondary antibodies (Jackson ImmunoResearch Laboratories, 1:500) and Alexa Fluor 488-conjugated secondary antibodies (Sigma, 1:500).

### Immunohistochemistry

Immunohistochemistry was performed as described previously^64^. Mouse eyecups were fixed in 4% paraformaldehyde (PFA) in phosphate-buffered saline (PBS) for 5 or 30 min. The tissue was then rinsed in PBS, cryoprotected with 30% sucrose in PBS, embedded in TissueTec OCT compound 4583 (Sakura), frozen, and sectioned. Frozen 20 μm sections on slides were dried for 30 min at room temperature, rehydrated in PBS for 5 min, incubated with blocking buffer (5% normal goat serum or normal donkey serum, and 0.1% Triton X-100 in PBS) for 1 h, and then with primary antibodies overnight at 4 °C. Slides were washed with PBS three times for 10 min each time and incubated with secondary antibodies for 2 h at room temperature. The specimens were observed under a laser confocal microscope (LSM700, Carl Zeiss).

### Toluidine blue staining

Retinal sections were rinsed with PBS and then stained with 0.1% toluidine blue in PBS for 1 min. After washing with PBS, slides were coverslipped and immediately observed under the microscope.

### Transmission electron microscopy

Transmission electron microscopic analysis was carried out as described previously^65^. Mouse eyecups were fixed with 2% glutaraldehyde, 2% PFA in 0.1 M phosphate buffer for 6 h on ice. After fixation with 1% osmium tetraoxide for 3 h, the retinas were dehydrated thorough a graded series of ethanol (30–100%) and propylene oxide. Finally, the retinas were embedded in epoxy resin. Sections were cut on an ultramicrotome (EM UC7, Leica) and stained with uranyl acetate and lead citrate. Retinal sections were observed by transmission electron microscope (H-7500, Hitachi). Synapses containing a single ribbon with horizontal cell processes (control) or space due to lack of horizontal cell dendrites (dHC) were selected for quantification.

### Multi-electrode array analysis

Mice were dark-adapted for more than 1 h before experiment and sacrificed. Eyes were enucleated under a dim red light. Under a stereomicroscope equipped with infrared (IR) image converter (C5100, Hamamatsu photonics) and IR illumination (HVL-IRM, Sony), the cornea and lens were ablated, and then the retina was isolated from the pigment epithelium. The retina was placed on a multi-electrode array (60pMEA200/30iR-Ti, Multichannel Systems: 60 electrodes, electrode size 30 × 30 μm, inter-electrode distance 200 μm) with ganglion cell layer facing down. The retina was continuously superfused with a solution bubbled with 95% O_2_ / 5% CO_2_ at the rate of 4 mL/min. The solution contained 116 mM NaCl, 10 mM D-glucose, 3.1 mM KCl, 24 mM NaHCO_3_, 0.5 mM KH_2_PO_4_, 2.5 mM CaCl_2_, 1 mM MgCl_2_, 1 mM Na-pyruvate, and 4 mg/L phenol red. The superfusate in the recording chamber was maintained at 35 °C. Each retina used in this study was obtained from different mice (note that we used 6 retinas from 6 control mice, 4 retinas from 4 dHC mice).

Spike discharges from retinal ganglion cells (RGCs) were amplified (MEA 1060-BC, Multichannel Systems) and stored at 20 kHz (PowerLab 16/35, AD Instruments) in computer. After band-pass filtering (100–3,000 Hz), the spikes were sorted into single unit activities by the principal component analysis and template matching with programs using MATLAB (Mathworks). Among several unit activities recorded from each electrode, we selected 1–3 single units with good signal-to-noise ratio for further analyses.

### Light stimulation and receptive field (RF) analysis

Light patterns were generated by Psychtoolbox3^66, 67^ on MATLAB, and the light stimulus presented on a cathode-ray tube display (S501J, refresh rate 60 Hz, 1,280 × 1,024 pixels, Iiyama) was projected onto the retina through optics. In Fig. 2, light stimulus on the retina was a square of 1,600 × 1,600 µm, and displayed for 2 s at 4.63 cd/m^2^. To evaluate the adaptation to light intensity (Fig. 5), the intensity of test flash stimulation varied between 3.85 and 13.27 cd/m^2^ (0.59~1.12 log/m^2^, corresponding to the “mesopic~photopic” level of mouse vision^55^). We applied the background illumination for more than 1 minute before the onset of the test flash. In Fig. 4, 2 s-drifting of grating from rostral to caudal side of the retina started after 2 s from the grating displayed. We also applied driftings to three other directions (to dorsal, to rostral, and to ventral side of the retina), and confirmed that these conditions yielded the same results statistically. The firing index (Supplementary Fig. 5b) was calculated as a ratio, (mean *R*_*during drifting*_ – mean *R*_*before stim*._)/(mean *R*_*during drifting*_ + mean *R*_*before stim*._). *R* is spike number. To screen the directional selectivity (DS) of RGC, (Supplementary Figs 6–9), we used a sinusoidal grating (0.15 cyc/deg, 1 Hz, 1.5 s, mean contrast, 60%, mean intensity, 3.26 cd/m^2^) that drifts in eight directions (0° ~ 360°, ∆45°; note that in one retina we used a sinusoidal grating that drifts in four directions: 0°, 90°, 180°, 360°). The direction selectivity index (DSI) was used to separate the DS- and non-DS-RGCs. DSI is calculated as |∑rd|/∑|rd|. rd denotes vector of spike number evoked in one directional drift. ∑|rd| denotes the sum of spike numbers evoked in eight directional drifts. |∑rd| denotes the vector sum of the eight directions. If the DSI of a RGC was higher than 0.3, the RGC was defined as direction selective. DS RGCs were separated based on velocity index (VI^42^, Supplementary Fig. 6). VI was calculated as (Resp_Fast_ – Resp_Slow_)/ (Resp_Fast_ + Resp_Slow_). Resp_Fast_ is the maximum response among the eight directions during fast motion, 1.2 mm/s. Resp_Slow_ is obtained during slow motion, 0.15 mm/s. If the VI of a RGC was a negative value, the RGC was defined as a slow-motion-tuning cell. The population in these retinas were consistent with the previous report: a majority of ON-DS RGC is tuned to slow motion, but many ONOFF-DS RGCs were tuned to fast motion^42^.

To estimate the spatiotemporal RF of RGCs, we used the reverse correlation method^35, 36^. The retina was stimulated with a series of pseudorandom (M-sequence) checkerboard patterns. Each frame (32 × 32 pixels, pixel size: 50 × 50 µm, mean intensity: 0.85 ~ 1.82 cd/m^2^) was updated at 30 Hz. After spike sorting, the spike-triggered average (STA) was calculated by the average of matrices of stimulus intensity, each of which preceded a spike evoked by a RGC. To obtain a spatial profile of RF, we set the threshold intensity for detecting the correlated region as mean + 6 SD of the noise intensity, which was calculated using the newly generated independent stimulus sequence. Then we determined the edges of the correlated region for 8 directions from the RF centers (0–315, *∆*45°). Spatial RF was defined as a 2D ellipse fitted to the 8 edges based on the least squares method. The RF area (*πab*) was computed from the major axis *a* and the minor axis *b* (Fig. 3f). To quantify the signal intensities of the RF center and surround, the RF center was defined as the pixel in which signal intensity of the RF was maximum. The surround index was calculated as the minimum intensity of pixels within 1 mm from the RF center (Fig. 3d,e).

### Data analysis

Peri-stimulus time histograms (PSTHs) were calculated with a 30-ms bin width in Fig. 2a, and 10-ms bin width in others. Response reliability (Fig. 2b, *left*) was quantified as an average of the Pearson’s correlation between light-evoked spike trains of each trial. CV of the inter-spike interval (Fig. 2b, *right*) was calculated as the SD divided by the mean of the inter-spike interval of light-evoked spikes. The response latency was defined as the time-to-response peak in PSTH after the stimulus onset (ON response) or offset (OFF response). Median of firing rate was calculated from the median of histograms of reciprocal of inter-spike interval during stimulation (2 s, ON response) or 2 s after stimulus offset (OFF response).

### OKR recording

The animal preparation, eye movement recording, and visual stimulus were as previously described in detail^68, 69^. Data were collected from control and dHC mice weighing 15.8–22.9 g. Each mouse was previously implanted with a head holder, which allowed the head to be fixed in the stereotaxic position during the experiments. Eye movement of the right eye of each mouse was measured using a video-based eye tracker system (Geteye, Matsuura-Denko-sha). The stimuli were presented on 19-inch computer monitors (spatial resolution, 1280 × 1024 pixels; refresh rate, 75 Hz; LCD, Mitsubishi, Tokyo, Japan). Three displays showing identical visual stimuli were set around the animal at the front and both sides. The visual stimuli were moving sinusoidal grating patterns (Michelson contrast, 64%; mean luminance, 100 cd/m^2^) generated using MATLAB (Mathworks, Natick, MA, USA) and with one of the five spatial frequencies selected randomly from a lookup table: 0.0313, 0.0625, 0.125, 0.25, or 0.5 cycles/deg in given trial. The temporal frequency was selected from 0.1875, 0.375, 0.75, 1.5, 3, 6, 12, or 24 Hz. At the beginning of each trial, a stationary visual pattern was presented, and then moved counterclockwise or clockwise at a constant speed for 100 ~ 200 ms (initial phase OKR), 1 ~ 30 s (late phase OKR; OKN). After a defined period, the pattern was removed.

### Statistics

In Figs 1j, 2b, 3f, 6c,d,g,h,k–n, Supplementary Figs 3a–c and 5a, *t*-test was used. In Fig. 4b, and Supplementary Fig. 6b,c, Chi-squared test was used and multiple comparisons were performed by adjusted standardized residual. In Fig. 5c,d, paired *t*-test was used with Bonferroni correction. In Figs2c,d, 5e, and Supplementary Figs 5b, 7, 8, Kolomogorov-Smirnov test was used. In Supplementary Fig. 9, One-way ANOVA was used. To perform mathematical fittings of functions, we evaluated the validity of the fittings by the least squares method. Error bars denote standard deviation (SD).

## Electronic supplementary material

Supplementary Information
