# Supplementary material for: Versatile functional roles of horizontal cells in the retinal circuit
Source: Sci Rep. 2017 Jul 17;7:5540. doi: 10.1038/s41598-017-05543-2 (PMC5514144; doi:10.1038/s41598-017-05543-2)
Supplement: Supplementary file 1 — Supplementary Information [file 41598_2017_5543_MOESM1_ESM.pdf]

Supplementary Information

Versatile functional roles of horizontal cells in the retinal circuit

Taro Chaya[1,4], Akihiro Matsumoto[2,4], Yuko Sugita[1], Satoshi Watanabe[1], Ryusuke Kuwahara[3], Masao Tachibana[2,*], Takahisa Furukawa[1,*]

[1]Laboratory for Molecular and Developmental Biology, Institute for Protein Research, Osaka University, JST, CREST, 3-2 Yamadaoka, Suita, Osaka, 565-0871, Japan.
[2]Department of Psychology, Graduate School of Humanities and Sociology, The University of Tokyo, JST, CREST, 7-3-1 Hongo, Bunkyo-ku, Tokyo, 113-0033, Japan.
[3]Research Center for Ultrahigh Voltage Electron Microscopy, Osaka University, 7-1 Mihogaoka, Ibaraki, Osaka, 567-0047, Japan.
[4]These authors contributed equally to this work.

[*]Correspondence should be addressed to T.F. (takahisa.furukawa@protein.osaka-u.ac.jp) or M.T. (tchbn2ms@l.u-tokyo.ac.jp).

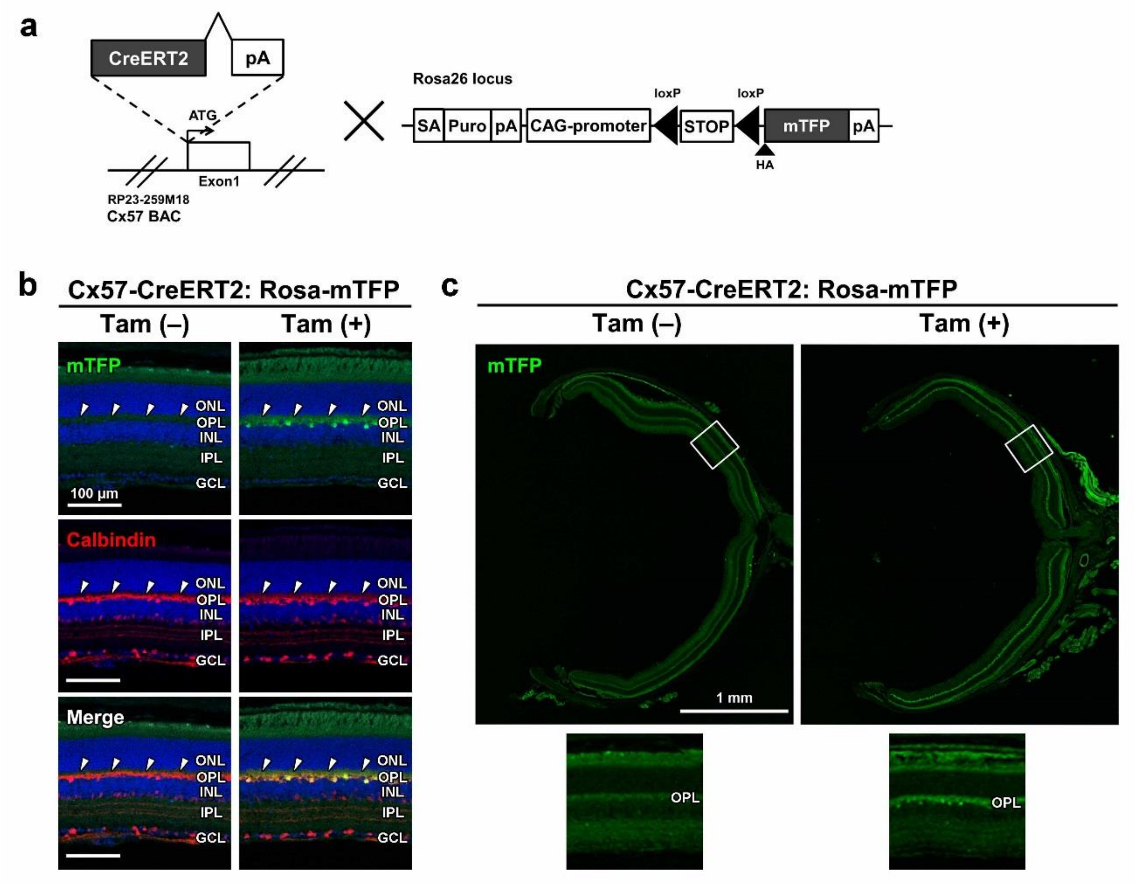

Supplementary Figure 1. ***Cx57*** **promoter induces gene expression specifically in horizontal cells in the retina.** (**a**) Schematic diagram of the *BAC-Cx57-CreERT2* construct, in which the *CreERT2-pA* cassette is integrated into the translation start site of the mouse *Cx57* gene, and *R26-CAG-LoxP-mTFP1* construct, in which the expression of Cre-mediated mTFP1 is induced under the control of the CAG promoter. (**b,c**) Immunohistochemical analysis of retinas in BAC-Cx57-CreERT2; R26-CAG-LoxP-mTFP1 mice with or without tamoxifen injection. mTFP1 signals were merged with those of Calbindin in the OPL (arrowheads in B). Enlarged images of boxed areas are shown in lower panels (**c**). Nuclei were stained with DAPI (blue). ONL, outer nuclear layer; OPL, outer plexiform layer; INL, inner nuclear layer; IPL, inner plexiform layer; GCL, ganglion cell layer.

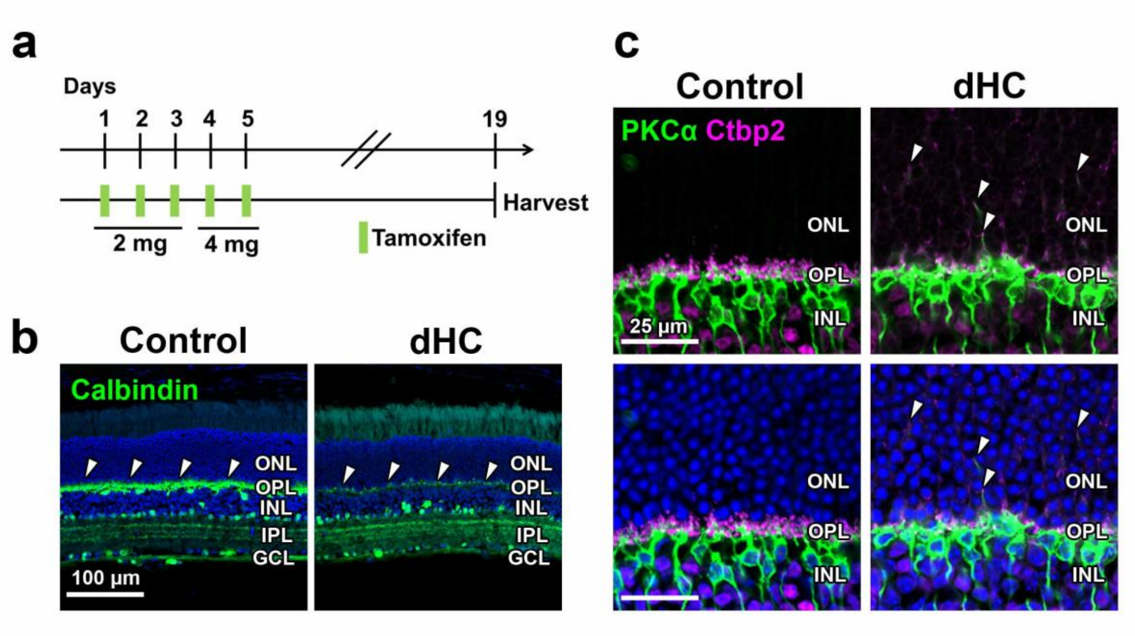

Supplementary Figure 2. Long-term effects of horizontal cell depletion on synaptic connections between photoreceptor and bipolar cells. (**a**) Schematic diagram of schedule for tamoxifen administration and harvest of retinas. (**b**) Retinal sections were immunostained with an anti-Calbindin antibody. Calbindin-positive cells in the OPL (HCs, arrowheads) were fragmented in the dHC mouse retina. (**c**) Retinal sections were immunostained using antibodies against PKCα (rod bipolar cell, green) and Ctbp2 (synaptic ribbon, magenta). Ectopic synapse formation was observed in the ONL of dHC retinas (arrowheads). Nuclei were stained with DAPI (blue). ONL, outer nuclear layer; OPL, outer plexiform layer; INL, inner nuclear layer; IPL, inner plexiform layer; GCL, ganglion cell layer.

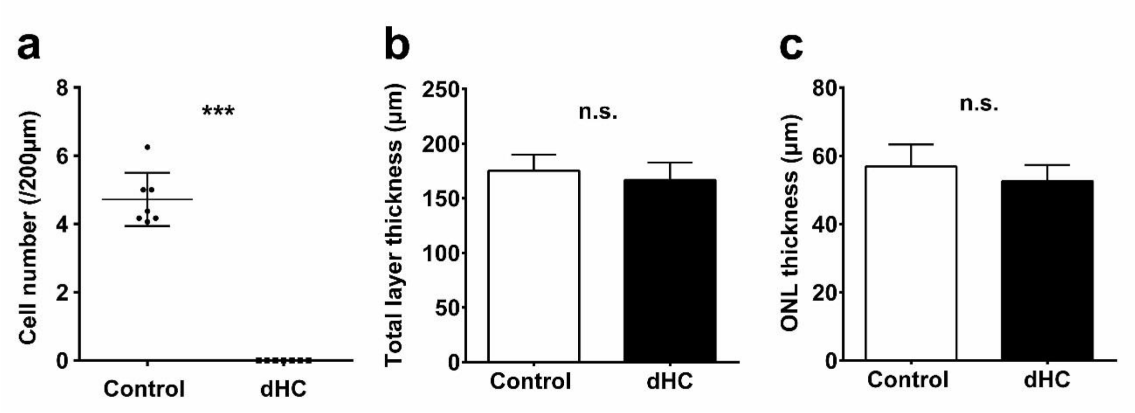

Supplementary Figure 3. Quantification of horizontal cell number and layer thickness in the dHC retina. (**a**) The number of HCs stained with the anti-Calbindin antibody was counted in control and dHC retinas (n = 7 retinas from seven animals for each genotype). At least two 320 µm areas in each retina were selected. (**b,c**) The thickness of the total retinal layer (**b**) and ONL (**c**) was measured in control and dHC mice (n = 7 retinas from seven animals for each genotype). Data are presented as mean ± SD. $p = 1.9\text{x}10^{-9}$ (**a**), $p = 0.33$ (**b**), $p = 0.18$ (**c**). ***$p < 0.001$. n.s., not significant.

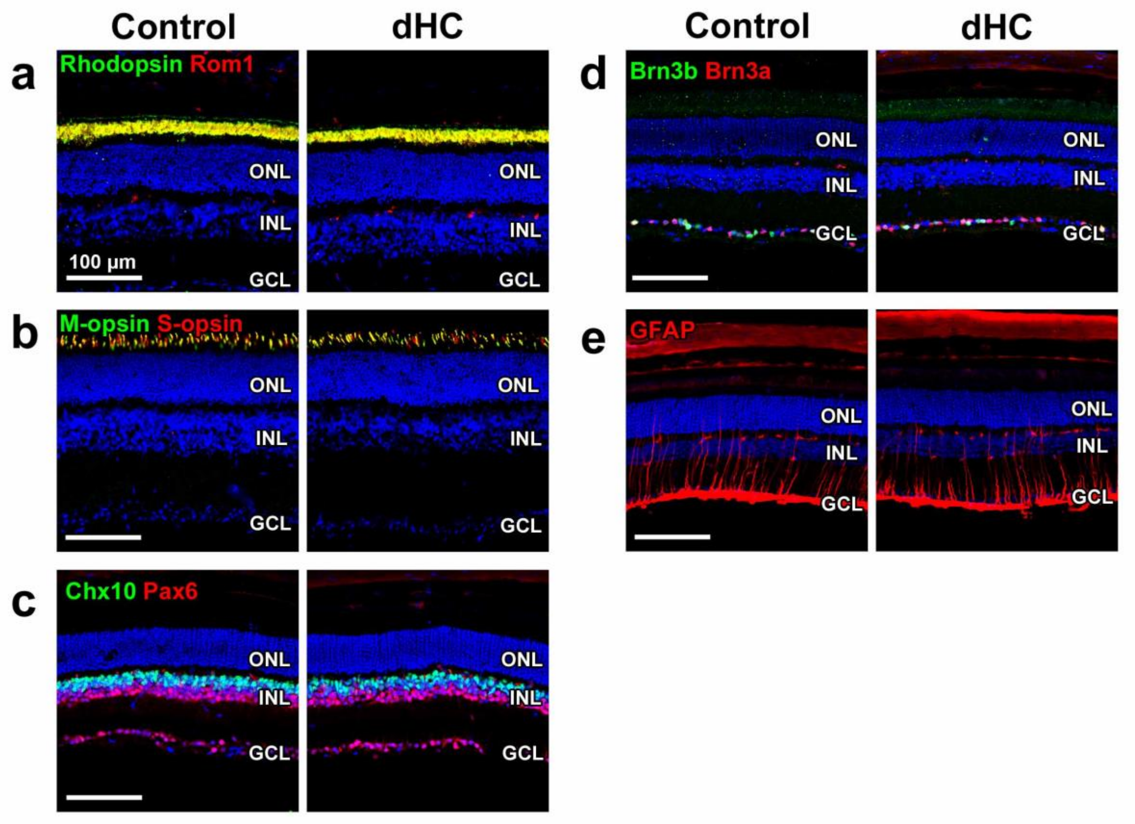

Supplementary Figure 4. Immunohistochemical analysis of major cell compositions in the dHC retina. (**a-e**) Retinal sections from control and dHC mice were immunostained with antibodies against Rhodopsin (a rod photoreceptor marker, green in **a**), RomI (a rod photoreceptor marker, red in **a**), M-opsin (a cone photoreceptor marker, green in **b**), S-opsin (a cone photoreceptor marker, red in **b**), Chx10 (a bipolar cell marker, green in **c**), Pax6 (a marker for amacrine and RGCs, red in **c**), Brn3b (an RGC marker, green in **d**), Brn3a (an RGC marker, red in **d**), and GFAP (a Müller glial cell marker, red in **e**). The number of each cell type was unaltered between control and dHC retinas. Nuclei were stained with DAPI (blue). ONL, outer nuclear layer; INL, inner nuclear layer; GCL, ganglion cell layer.

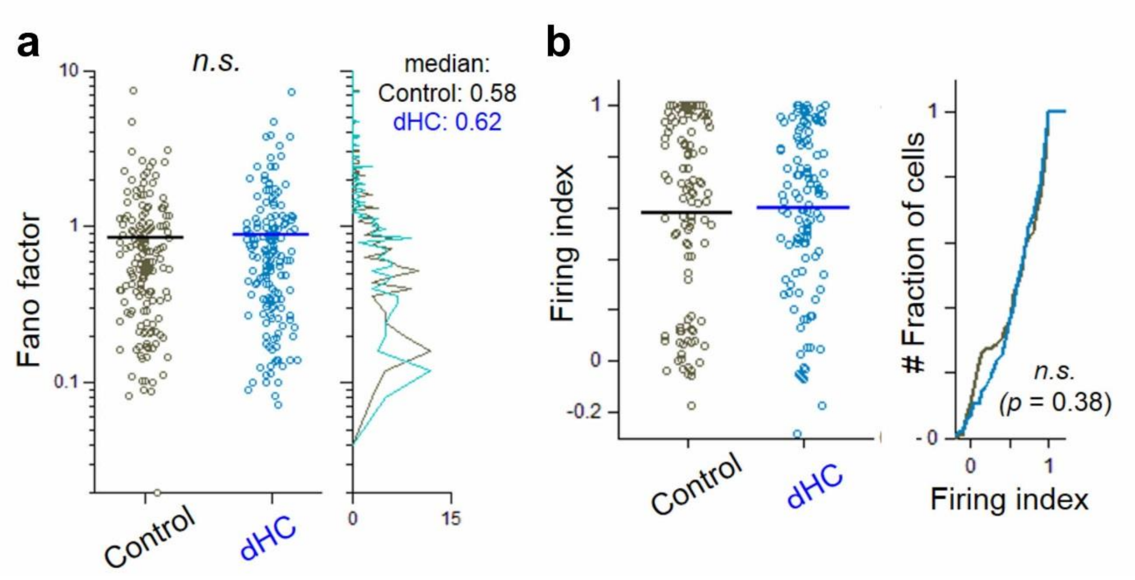

Supplementary Figure 5. Firing characteristics of RGCs during light stimulation. (**a**) Left, Fano factor (FF) of each RGC was calculated as a measure of the trial-to-trial variability of spike numbers. Spike train during light stimulation of each trial was binned at 40 ms intervals, and FF was calculated as the variance of the spike number divided by the mean. Horizontal bar, mean (0.84 in control, 0.88 in dHC). Unpaired *t*-test, $p = 0.68$. Right, histograms were calculated with 0.04 bin. Gray, control; cyan, dHC. 186 RGCs in 6 control retinas. 136 RGCs in 4 dHC retinas. (**b**) Left, Firing index (FI) was calculated as a measure of the response during drifting grating (Methods). FI 0 denotes no response modulation by stimulation. Horizontal bar, mean (0.58 in control, 0.59 in dHC). Right, cumulative distribution of FI. Kolmogorov-Smirnov test, $p = 0.38$. 159 RGCs in 4 control retinas. 161 RGCs in 4 dHC retinas.

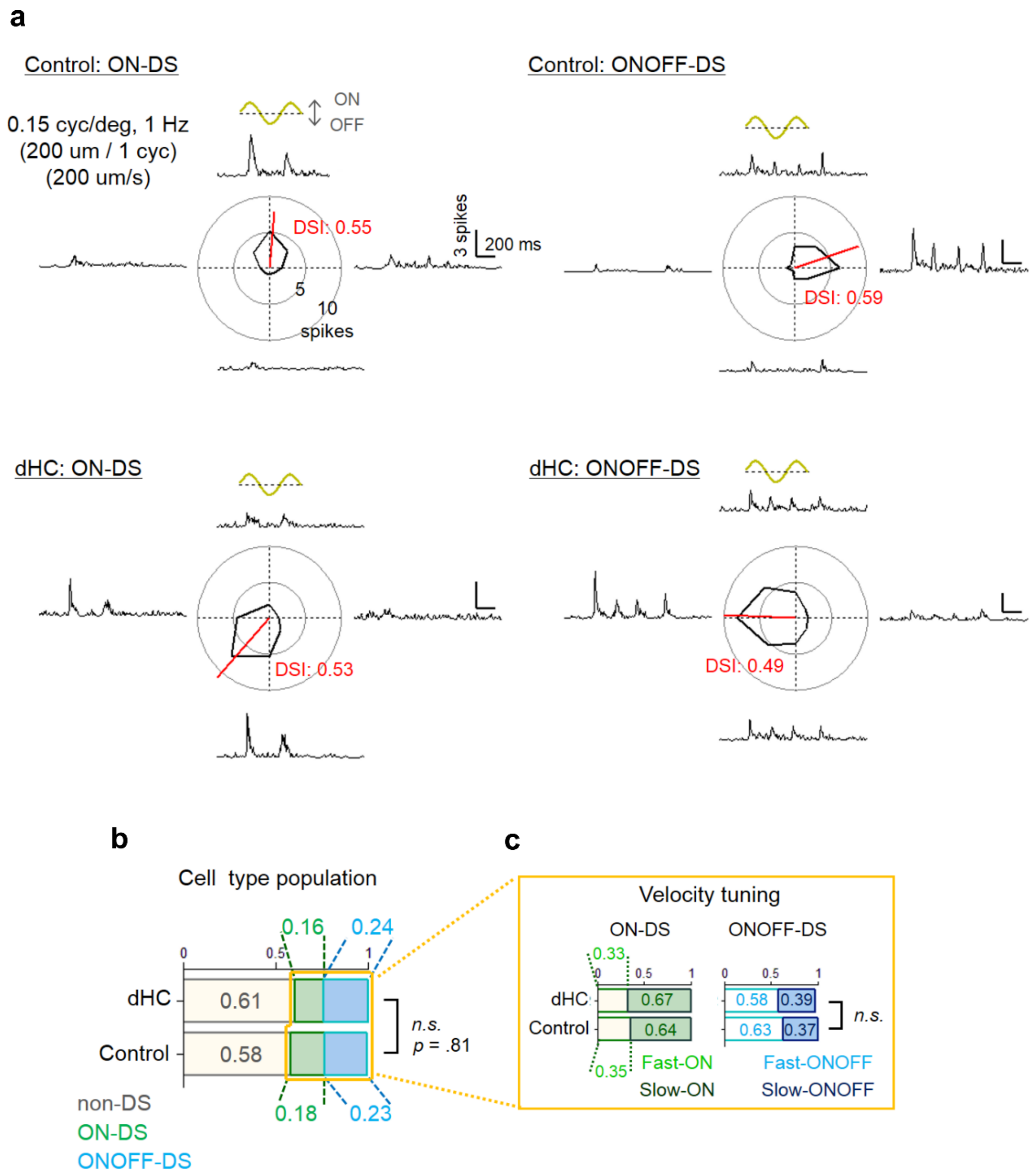

Supplementary Figure 6. Direction selectivity was maintained in the dHC retina. (**a**) Direction selective RGCs (DS-RGCs) in the control (upper) and dHC (lower) retinas. Left, ON-DS RGCs. Right, ONOFF-DS RGCs. Black trace, responses (peri-stimulus time histogram, 3 trials) to a sinusoidal grating (yellow, a sinusoidal grating (0.15 cyc/deg, 1 Hz, 1.5 s, mean contrast, 60 %, mean intensity, 3.26 cd/m^2) that drifts to one of four directions (0° ~ 360°, Δ90°, see Methods). Polar plot shows the spike number (black) and the direction selective index (DSI, red, see Methods). (**b**) RGC-type

population in the ontrol (upper) and dHC (lower) retinas. 151 RGCs in 6 control retinas. 134 RGCs in 4 dHC retinas. The proportion of RGC types (non-DS, ON-DS, and ONOFF-DS RGCs) was not different between control and the dHC retina (Chi-squared test, p = 0.81). (**c**) Proportion of the Fast-motion-tuned and Slow-motion-tuned DS RGCs. DS RGCs were separated based on the velocity index (VI, see Methods). The proportion of Fast-motion-tuned and Slow-motion-tuned RGCs was not different in ON-DS (Chi-squared test, p = 0.86) and ONOFF-DS (Chi-squared test, p = 0.77) RGCs. n.s., not significant.

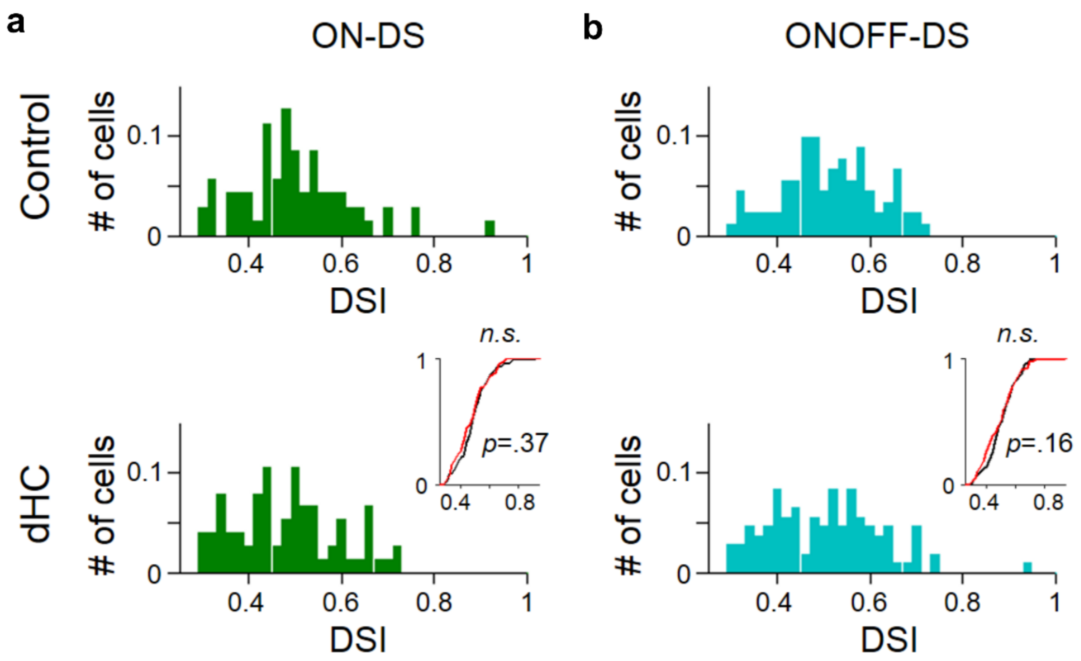

Supplementary Figure 7. HC depletion did not change the direction selective index of DS RGCs. (**a,b**) Distribution of DSI of ON-DS (a) and ONOFF-DS (b) RGCs in the control (upper) and dHC (lower) retinas. Inset, cumulative distribution of DSI. black, control. red, dHC. 28 ON-DS RGCs in 6 control retinas, 21 ON-DS RGCs in 4 dHC retinas, Kolmogorov-Smirnov test, p = 0.37. 35 ONOFF-DS RGCs in 6 control retinas, 32 ONOFF-DS RGCs in 4 dHC retinas, Kolmogorov-Smirnov test, p = 0.16. n.s., not significant.

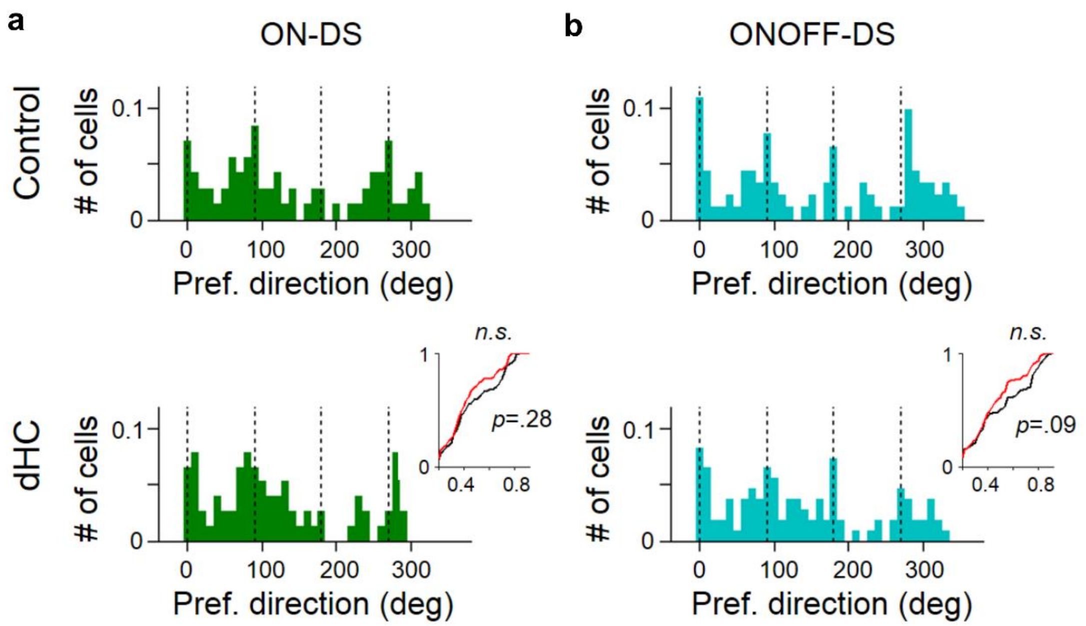

Supplementary Figure 8. HC depletion did not change the preferred direction of DS RGCs. (**a,b**) Distribution of preferred direction of ON-DS (a) and ONOFF-DS (b) RGCs in the control (upper) and dHC (lower) retinas. Preferred direction was defined as the motion direction that evoked the maximum response. black dotted line, 0°, 90°, 180°, 270°. Inset, cumulative distribution of DSI. black, control. red, dHC. 28 ON-DS RGCs in 6 control retinas, 21 ON-DS RGCs in 4 dHC retinas, Kolmogorov-Smirnov test, p = 0.28. 35 ONOFF-DS RGCs in 6 control retinas, 32 ONOFF-DS RGCs in 4 dHC retinas, Kolmogorov-Smirnov test, p = 0.09. n.s., not significant.

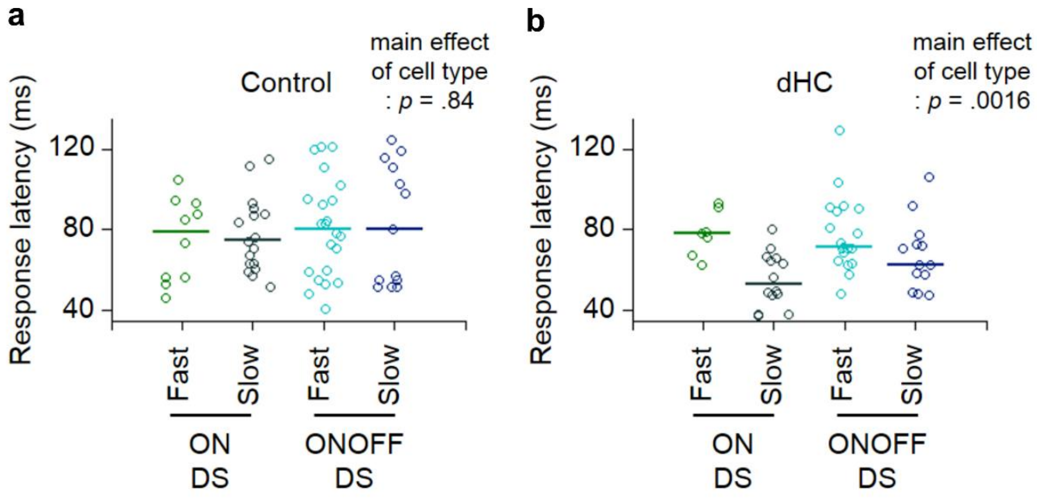

Supplementary Figure 9. ON Response latency in four DS-RGC types. (**a,b**) Response to the flash stimulation (1.6 x 1.6 mm, 2 s, 4.63 cd/m^2) in control (a) and dHC (b) retinas. The response latency was defined as the time-to-response peak in the peri-stimulus time histogram after the stimulus onset. horizontal bar, mean latency of each type. Difference between the control and dHC retinas was distinctive in the Slow-motion-tuned ON-DS RGCs. Control (6 retinas): 28 ON-DS (10 Fast-motion-tuned, 18 Slow-motion-tuned) RGCs, 35 ONOFF-DS (22 Fast-motion-tuned, 13 Slow-motion-tuned) RGCs, One-way ANOVA, main effect of cell type, $p = 0.84$. dHC (4 retinas): 21 ON-DS (7 Fast-motion-tuned, 14 Slow-motion-tuned) RGCs, 32 ONOFF-DS (19 Fast-motion-tuned, 13 Slow-motion-tuned) RGCs, One-way ANOVA, main effect of cell type, $p = 0.0016$.
